# The importance of power in photobiomodulation: a systematic review and meta-analysis of high-intensity laser therapy

**DOI:** 10.3389/fnint.2026.1914223

**Published:** 2026-09-14

**Authors:** William Todd Penberthy, Charles E. Vorwaller

**Affiliations:** 1 Personalized Health Research Services, LLC, Winter Park, FL, United States; 2 Aspen Lasers, LLC, Lindon, UT, United States

**Keywords:** photobiomodulation therapy, high-intensity laser therapy, class IV laser therapy, transcranial photobiomodulation, laser power, musculoskeletal pain, systematic review and meta-analysis, low-level laser therapy

## Abstract

**Introduction:**

Low-level laser therapy (LLLT; ≤ 0.5 W) is guideline-recommended in the United States for shallow-tissue oral mucositis and other superficial pathologies but has generally failed in trials for deeper tissue conditions due to limited penetration and subtherapeutic dosing. By contrast, Class IV high-intensity laser therapy (HILT; ≥ 0.5–80 W), typically at ∼100-fold higher power, has proven successful for many of these deeper tissue indications.

**Methods:**

We systematically searched PubMed, Google Scholar, and the Cochrane Database for Class IV HILT trials published from 2018 to April 2026, classifying interventions as effective when they showed large effect sizes (Cohen’s *d* > 0.8).

**Results:**

Across 105 included studies (*n* = 2,710 HILT; *n* = 2,925 controls), no serious adverse events were reported. HILT reduced pain in all randomized trials of knee (*k* = 27), spine/neuromuscular (*k* = 20), shoulder/arm (*k* = 20), and wounds (*k* = 2), with > 90% showing large effect sizes. Additional benefits were observed in disorders of the feet (*k* = 13), hand/wrist (*k* = 8), face/jaw (*k* = 7), osteoporosis (*k* = 2), endometriosis, coronary artery disease, cognitive impairment, fibromyalgia, COVID-19, and obesity. HILT yielded durable analgesia, with large VAS pain reductions at end of treatment, < 3-month, and ≥ 3-month follow-ups (SMD -0.98, -1.18, and -1.01; *k* = 50, 19, and 21, respectively). Functional outcomes improved consistently, including range of motion (92%; *k* = 26), functional performance (84%; *k* = 57), and quality of life (85%; *k* = 25). Structural and physiological changes were observed, including increased knee cartilage thickness, reduced rotator cuff tear size, weight loss, and improved nerve conduction. Comparative trials showed HILT produced greater pain reduction than LLLT in 13/13 studies, all with large effect sizes. Mean HILT power was 9.3 W (median 10.0; range 0.5–30), and mean power density was 19.5 W/cm^2^ (median 10; range 0.1–52.5).

**Conclusion:**

Class IV high-intensity laser therapy appears effective and safe for durable pain reduction across deep-tissue musculoskeletal pathologies, supporting its recognition in clinical practice and coverage by payers as a valuable therapeutic option. Further multicenter trials are needed to refine treatment protocols.

**Systematic review registration:**

https://www.crd.york.ac.uk/PROSPERO/view/CRD420251021772, identifier CRD420251021772.

## 1 Introduction

Since the inception of photobiomodulation therapy (PBMT) over 50 years ago, PBMT has primarily been used to treat shallow tissue pathologies (<1 cm deep) using low-powered light (<500 mW). Numerous therapeutic successes and countless basic scientific studies have demonstrated the advantageous properties and clinical benefits of PBMT for a growing range of shallow tissue pathological indications. However, when attempting to treat deeper tissue pathologies, the lower-powered approaches have consistently failed to yield positive results due to the light’s inability to penetrate the deeper pathological tissues (Calderhead, 2017; Google Docs, 2023 Photobiomodulation (PBM) research—a comprehensive database, n.d.). Over the past two decades, since the first FDA approval of higher-powered coherent laser therapy in the range of 1–80 watts (W), numerous studies investigating higher powers for the treatment of deep tissue pathologies have demonstrated positive outcomes for the same indications (Alayat M. S. et al., 2018; Song et al., 2018; Wyszyńska and Bal-Bocheńska, 2018; Alayat et al., 2019; Ezzati et al., 2020b; Ahmad et al., 2022; Al-Shenqiti, 2022; Starzec-Proserpio et al., 2022). This is commonly referred to as high-intensity laser therapy (HILT). The full range of indications that respond positively to HILT is currently in a discovery phase of research and an area of focus for this meta-analysis. Our meta-analysis is accompanied by our review highlighting pioneering firsts in HILT clinical research, both historical and ongoing.

### 1.1 PBMT discovery, acceptance, and the general limitations of LLLT to treating shallow tissue pathologies only

Photobiomodulation therapy (PBMT) was first observed in 1967, when red light irradiation unexpectedly increased hair growth in shaved mice without causing cancer (Mester et al., 1968). In the early 1970s, Andrew and Adam Mester applied low-power helium–neon laser light (10 mW, 630 nm) to recalcitrant non-healing leg ulcers, achieving complete recovery in 823 of 1,018 patients (81.4%), with almost 74% of long-standing non-healing ulcers closing within about 5.5 months (Mester et al., 1972; Mester and Mester, 1989). These results underscore the clinical potential of PBMT, particularly given that 5-year mortality for patients with diabetic and ischemic foot ulcers exceeds that of several common cancers (Armstrong et al., 2020).

Recognition of PBMT accelerated after the development of the “NASA LED” in Professor Harry Whelan’s Space Medicine laboratory in 1998, which enabled affordable, energy-efficient, and widely accessible light sources suitable for home-based therapies (Kim and Calderhead, 2011). By 2020, robust evidence for low-level laser therapy (LLLT) in oral mucositis led to physician consensus guidelines and insurance coverage for PBMT at powers of approximately 25–150 mW (Zadik et al., 2019; Aetna, 2026 Cold Laser and High-Power Laser Therapies—Medical Clinical Policy Bulletins | Aetna, n.d.). Evidence-based PBM guidelines have subsequently expanded to include multiple superficial conditions, such as androgenic alopecia, decubitus ulcers, pain associated with diabetic foot ulcers, acute radiation dermatitis, and peripheral neuropathy (Maghfour et al., 2025).

However, the efficacy of LLLT at these low powers is largely confined to shallow tissues less than about 2 cm deep. Controlled trials using PBMT below 500 mW for deeper musculoskeletal pathologies have repeatedly failed to show meaningful benefit (Huang et al., 2015; Ordahan et al., 2018; Ezzati et al., 2019; Kaydok et al., 2020; Naruseviciute and Kubilius, 2020; Vassão et al., 2020). Biophysical analyses demonstrate that such low-power light cannot deliver therapeutic irradiance beyond roughly 2 cm, a depth insufficient for most musculoskeletal arthropathies, which typically involve deeper joint and periarticular structures (Simpson et al., 1998; Esnouf et al., 2007; Henderson and Morries, 2015).

### 1.2 High-powered lasers confer benefits to deep tissue pathologies

On the other hand, high-intensity laser therapy (HILT; higher power) used in clinical practice can penetrate deeply into tissues to deliver effective levels of photobiomodulation therapy. Research by Morries et al. demonstrated that a 15 W laser with a 7 cm^2^ spot size can penetrate 3 cm (1.2 inches) of tissue while maintaining an established therapeutically active power density of approximately 50 mW/cm^2^ (Henderson and Morries, 2015). As a result, the use of an 80 W laser enables the delivery of therapeutic doses of PBMT to tissues as deep as 15 cm (6 inches) (White et al., 2019; Ahmad et al., 2022). Multiple independent studies have shown that HILT consistently produces positive outcomes, particularly in the treatment of knee osteoarthritis.

**Definitions**
**Power:** The rate at which energy is emitted from a laser expressed in watts (W).
**Power density (irradiance or energy density):** The quotient of the incident laser power expressed as watts per cm2 (W/cm^2^). The spot size is critical. A 1W powered laser is therapeutic (10-100W/cm^2^) and athermal if the spot size is 10 cm^2^ (100W/cm^2^), but this same 1W becomes surgical if the spot is focused to a 100ţm diameter (13,000W/cm^2^).
**Energy density or fluence (“dose”):** The amount of energy within light expressed in joules per area (J/cm2).
**Pulse:** The duration of time the light is on. This most typically ranges from milliseconds to nanoseconds, e.g., 100 ţs.

The FDA class IV device designation encompasses *therapeutic* lasers since it continues to be generally defined by powers as low as 500 mW even though the class IV designation has historically been used to *primarily* refer to *surgical* lasers, which use *higher* powers (e.g., 100–300 W) and *longer* wavelengths (e.g., 10,600 nm CO_2_ lasers or 2,940 nm Er: YAG lasers) than *therapeutic* lasers. This *surgical* laser light is highly absorbed by water to cause flash-boiling, ablation, or carbonization. By contrast, HILT light is not appreciably absorbed by water due to shorter 800–1,064 nm wavelengths and lower power settings commonly ranging from 2 to 30 W, which impart nondestructive *therapeutic* cellular activities in a painless fashion and ultimately penetrates to deeper tissues because of the wavelength (Calderhead, 2017). A 50 W CO*2* surgical laser becomes therapeutic when defocused to a 1Z cm^2^ spot size. Thus, spot size alone can determine whether a given light is surgical or therapeutic and also governs penetration depth at a fixed wavelength and collimation (Calderhead, 2017). Ultimately, the power density (irradiance; W/cm^2^) is the most informative parameter controlling the depth of penetration of light for a common specific wavelength.

The FDA only recognized clearance for class IV *therapy* laser devices as recently as 2003, Avicenna (Avicenna Laser Technology, Inc, 2023). Several medical associations have recommended high-powered photobiomodulation therapies. In 2021, the American Academy of Orthopedic Surgeons began recommending laser therapy for the treatment of osteoarthritis of the knee joint (Brophy and Fillingham, 2022). The American College of Physicians issued a guideline for treating non-radicular low back pain with PBMT starting in 2017 (Qaseem et al., 2017). The American Physical Therapy Association Orthopedic Section began issuing PBMT treatment recommendations for heel pain, plantar fasciitis, neck pain, and Achilles tendinitis (Carcia et al., 2010; Martin et al., 2014; Blanpied et al., 2017). The World Health Organization now recommends laser therapy and its photobiomodulation benefits for neck pain (Haldeman et al., 2008). The list of recommended treatment indications continues to expand.

Photobiomodulation therapy has been established and accepted at all levels for treating shallow tissue pathologies. However, the most commonly used power settings have been limited to lower powers (≤ 500 mW; class III), which have generally failed to produce positive outcomes when treating deep tissue pathologies (> 2 cm). By using high-powered (0.5–80 W) laser PBMT, positive outcomes have been repeatedly achieved due to the ability of high-powered lasers to penetrate depths of up to 15 cm (Henderson and Morries, 2015; White et al., 2019; Ahmad et al., 2022). More medical professionals in numerous fields of study are becoming aware of the possibilities of using higher-powered laser PBMT to reach deeper tissues.

The primary objective of this meta-analysis was to begin to assess the efficacy of high-powered laser PBMT for categorical indications as limited to *controlled* clinical trial data. This includes considering a wide range of outcome metrics, including pain reduction, range of motion increases, functionality improvements, quality of life improvements, wound closures, neurophysiological measures, and others, along with consideration of adverse events as a measure of safety. The incidence of adverse effects was also assessed as a measure of safety. The secondary objective was to perform a summary analysis of the optimal laser parameters (power settings, dosages, and wavelengths) and treatment plans for each indication based on available controlled trial data. Accordingly, a suggested summary table of optimal laser parameters and treatment plans is offered at the end of the results section.

## 2 Methods

### 2.1 Search for evidence and article selection

This review followed PRISMA 2020 and Cochrane guidelines (Page et al., 2021). This protocol was registered on PROSPERO under CRD420251021772 on March 31st, 2025. The population, intervention, comparison, and outcome (PICO) question was, “Which indications have demonstrably responded with positive outcomes in RCTs that have evaluated the use of HILT as a treatment?” The following components were considered: Population: Any diagnosed indication that was treated with HILT; Intervention: HILT; Comparison: Either placebo as non-therapeutic guiding red light, LLLT, or an alternative treatment; Outcome: Measures of pain, function, disability, quality of life, range of motion, nerve conductivity or action potential, wound assessment, bone density, grip strength, cartilage volume, and/or tendon tear. The study selection process is summarized in Figure 1, which presents the PRISMA flow diagram for records identification, screening, eligibility assessment, and final inclusion.

**FIGURE 1:**
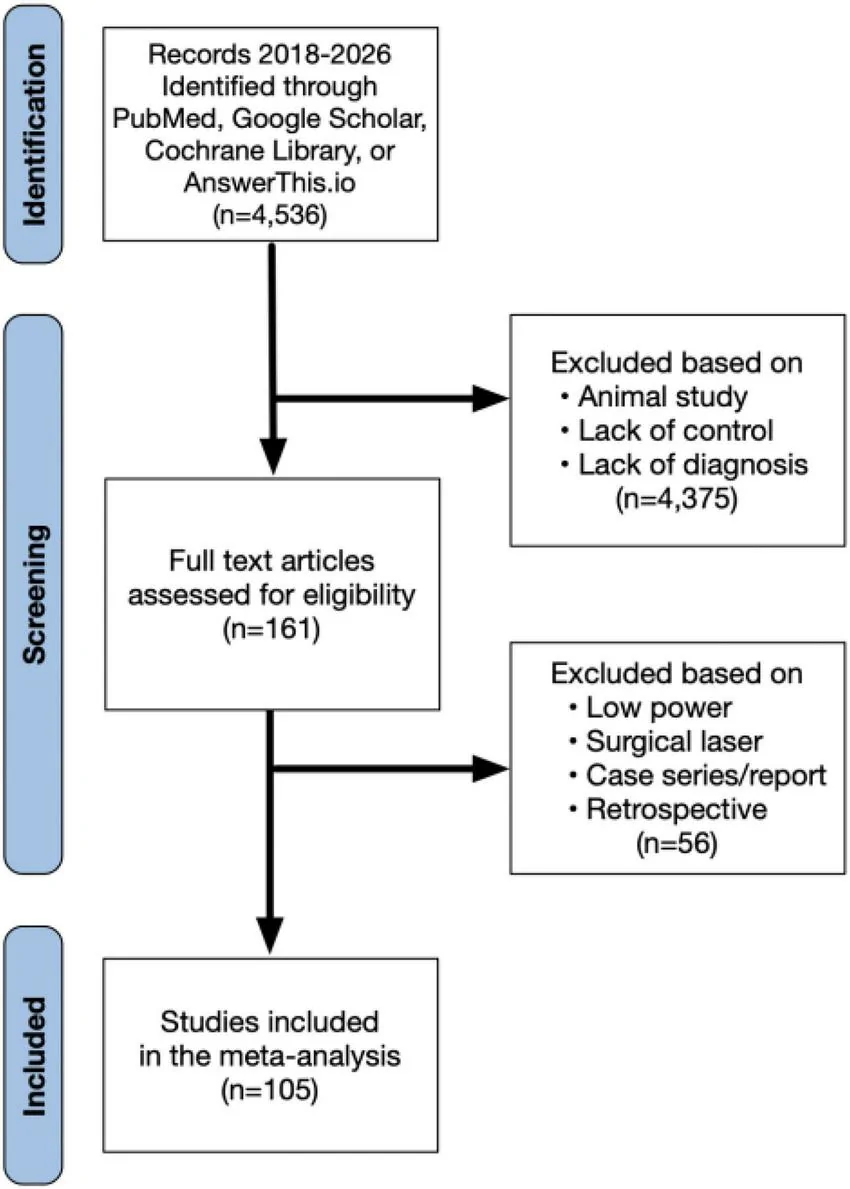
Preferred reporting items for systematic reviews and meta-analyses (PRISMA) flowchart illustrating the data retrieval protocol used in this work.

We conducted a systematic search of MEDLINE (PubMed), Embase, Cochrane Central Register of Controlled Trials (CENTRAL), Web of Science, Scopus, and Google Scholar for articles published from January 1, 2018 to May 1, 2026. The search combined database-specific controlled vocabulary with free-text terms for high-intensity or high-power laser photobiomodulation (for example, “high-intensity laser therapy,” “high-power laser,” “HILT,” “Nd:YAG”) together with terms for randomized or controlled clinical trials. No restrictions were applied on diagnoses at the search stage; we considered trials in which high-intensity laser therapy was used to treat any clinically diagnosed condition in human participants.

In addition, we screened the reference lists of all included trials and relevant reviews and used forward citation tracking in Web of Science and Google Scholar to identify further randomized or controlled trials. As a supplementary step, we used an AI-assisted literature tool (AnswerThis.io) to query phrases related to high-intensity/high-power/class IV laser photobiomodulation in controlled clinical trials addressing any diagnosed indication.

Records were cross-checked against the original publications and then passed through the same eligibility screening and data extraction procedures as records retrieved from the bibliographic databases. Duplicates identified across databases were removed, after which records were grouped by author and by target anatomy to resolve any remaining within-category duplicates. We then excluded studies with duplicated or incomplete data.

### 2.2 Inclusion and exclusion criteria

The inclusion criteria were as follows: (1) controlled clinical trial studies, (2) published between 2018 and May 2026, (3) use of higher-powered lasers with average power settings > 0.5 W, (4) patients with a medical diagnosis, (5) comparison of higher-powered lasers versus LLLT or placebo lasers or a common background, and (6) the use of outcome measures such as pain, range of motion, quality of life, cartilage thickness, disability indices, bone density, or gait. The exclusion criteria included (1) fundamental research or studies conducted on animals, (2) controlled trials involving patients without a medical diagnosis, (3) review articles, and (4) articles lacking statistical and clinical data. For publications that only provided information on peak power, frequency, and pulse time, the authors calculated the average power using the formula (Huang et al., 2011): average power = peak power x hertz frequency x pulse duration.

### 2.3 Data extraction

The following outcome measures were extracted from controlled trials: pain (Visual Analog Scale, VAS; Numerical Pain Rating Scale, NPRS, MPQ, SPADI, WOMAC), range of motion (ROM; rotation; flexion), quality of life (Short Form-36, SF-36; Nottingham Health Profile, NHP; European Quality of Life, EuroQoL), function (shortened version of Disabilities of the Arm, Shoulder, and Hand, QuickDASH; Constant-Murley Score, CMS; Shoulder Pain and Disability Index, SPADI; Functional Independence Measure, FIM; Endometrial Health Profile-5, EHP-5; lung for COVID-19; Foot and Ankle Outcome Score, FAOS), disability index (Neck Disability Index, NDI; Oswestry Disability Index, ODI), ultrasound structural improvements (knee cartilage space; rotator cuff tear size), wound assessment (size; appearance; Bates-Jensen Wound Assessment Tool, BWAT; Pressure Ulcer Scale for Healing, PUSH), swelling, endometriosis adhesions, walking (GAITRite; distance), smiling (Bell’s Palsy related), eyebrow movement (Bell’s Palsy related), ICU admissions (COVID-19 related), mortality (COVID-19 related), bone density, strength (hand grip; shoulder), nerve conductance, and action potential (Compound Muscle Action Potential, CMAP). Interventions and sample size, evaluation time, and country conducting the study were also summarized. For HILT application methods, the output power (watts, W), energy density (irradiance, W/cm^2^), wavelength, pulse settings (pulse time in μs; frequency, Hz) type of laser, dosage (joules, J, or J/cm^2^ when available), process of application, application times, number of treatment sessions per week, number of weeks of treatment, and outcome times were extracted when described in the text for analysis of laser settings achieving best outcomes. The characteristics of study designs were documented to include a description of comparators, such as placebo or alternative treatments, and the level of blinding employed (single, double, or triple). These factors were considered important measures to assess potential bias in the studies.

### 2.4 Statistical analysis

The significance level was based on reported *p* scores < 0.05. Confidence intervals were set to 95% (CI 95%) for all variables. The statistical method was designed to specifically measure the effect size (ES) attributable to HILT compared to the control group. To evaluate the magnitude of the difference between groups, the authors determined the effect size of each variable using Cohen’s *d* (Cohen, 1988; Brand et al., 2011). The standardized mean of group assessments made before and after treatment was divided by the pooled standard deviation. Differences in effect sizes between the HILT and control groups was calculated by subtracting the effect size for each group for determining the relative efficacy specifically attributable to HILT (Ezzati et al., 2020b).


$$E\,S=\frac{M\,t\,i\,m\,e\,z\,e\,r\,o-M\,f\,o\,l\,l\,o\,w\,u\,p}{S\,D\,p\,o\,o\,l\,e\,d}$$

Differenceineffectsize=ES-1ES2

Interpretation of effect sizes followed Cohen’s thresholds: small ≤ 0.2, medium 0.2–0.8, and large > 0.8 (Cohen, 1988). HILT’s post-treatment analgesic effects in knee, spine/back, shoulder, and HILT-vs.-LLLT studies were summarized as standardized mean differences (SMDs) and pooled using inverse-variance weighting. Heterogeneity was quantified with the Chi^2^-based I^2^ statistic, with values of 25, 50, and 75% indicating low, moderate, and high heterogeneity, respectively, and presented as forest plots (Fekete and Gyõrffy, 2025).

Power density (W/cm^2^) is the key determinant of penetration depth at a given wavelength, collimation, and duty cycle, yet it is often not reported in PBMT studies. Because spot size directly sets power density, the same device can be surgical when tightly focused and therapeutic when defocused; for example, a surgical CO*2* laser can become therapeutic when its beam is broadened (Calderhead, 2017). Given our focus on penetration depth, we report power density throughout this meta-analysis whenever spot size is described and can be calculated from the original studies.

A methodological limitation across many included trials was incomplete reporting of spot size, which prevented calculation of power density in a subset of studies. Because penetration depth and tissue dosing are governed primarily by power density (irradiance), rather than device power alone, this under-reporting obscures true dose-response relationships between high power density and clinical benefit.

### 2.5 Assessment of risk of bias and certainty

To assess the risk of bias in the included randomized controlled trials, we followed Cochrane recommendations and used the robvis (Risk-Of-Bias VISualization) tool (McGuinness and Higgins, 2021). Five domains were evaluated (randomization process, deviations from intended interventions, missing outcome data, measurement of outcomes, and selective reporting), and each study was rated as having low, moderate, or high risk of bias in each domain.

We then assessed the overall certainty of evidence for each meta-analytic outcome using the Grading of Recommendations Assessment, Development and Evaluation (GRADE) approach (Balshem et al., 2011) For each outcome, certainty was judged across five domains: risk of bias, inconsistency, indirectness, imprecision, and publication bias. Starting from high certainty for randomized trials, we downgraded the certainty rating by one or more levels (to moderate, low, or very low) when serious or very serious limitations were identified in any domain.

## 3 Results

### 3.1 Study selection

A total of 105 studies met the inclusion criteria for this meta-analysis (Figure 1). These studies most commonly assessed pain followed by physical function, range of motion, quality of life, strength, and anatomical changes (Tables 1, 2). Studies were primarily conducted in Turkey (*k* = 31) and Egypt (*k* = 22), with smaller numbers in Iran (*k* = 13), India (*k* = 10), Saudi Arabia (*k* = 7), China (*k* = 6), Thailand (*k* = 4), United States (*k* = 3), Indonesia (*k* = 3), Spain (*k* = 2), Italy (*k* = 1), and other countries with a single study (Lithuania, Thailand, Malaysia, Portugal, Poland; Table 2).

**TABLE 1:** Summary of RCT Outcomes and Key Findings: Pain, Function, ROM, and QoL.

Knee						
**Reference**	**Blinding**	**Key findings**	**< Pain**	**> ROM**	**>QoL**	**> Function**
Knee osteoarthritis (Ahmad et al., 2023)	2	Pain: NPRS scale flexion reduced	**+**	**+**	**+**	**+**
Knee osteoarthritis (Akaltun et al., 2020)	2	Cartilage thickness increased	**+**	**+**		**+**
Subacromial impingement syndrome (Akbaş et al., 2025)	2	Pain reduced function: SPADI	**+**			**+**
Knee osteoarthritis (Anupma et al., 2025)	2	Pain reduced	**+**			
Knee osteoarthritis (Astri et al., 2022)	2	Increased function 50-ft walk test	**+**			**+**
Knee osteoarthritis (Bettencourt, 2020)	1	Pain reduced at EOT and 1 mo after cessation	**+**			
Knee osteoarthritis (Ceyhan and Karaca, 2025)	?	Improved functional walking fast tests, getting up/down, stair climb	**+**		**+**	**+**
Knee osteoarthritis (Ciplak et al., 2018)	1	Pain reduced function: WOMAC	**+**			**+**
Knee osteoarthritis (Ekici and Ordahan, 2023)	2	Cartilage thickness increased	**+**	**+**		**+**
Hemophilic arthropathy (El-Shamy and Abdelaal, 2018) Egypt; 27976591	2	Gait performance improved	**+**		**+**	**+**
Juvenile rheumatoid arthritis (El-Shamy et al., 2018)	2	Gate improved: GAITRite	**+**			**+**
Knee osteoarthritis (Ezzati et al., 2024)	1	Function improved at 1 mo follow-up	**+**			**+**
Knee osteoarthritis (Koevska et al., 2025)	1	Functional ability increased stiffness decreased	**+**	**+**		**+**
Knee osteoarthritis (Laotammateep et al., 2025)	2	All patients improved	**+**			-
Knee osteoarthritis (Mostafa et al., 2022)	2	Gate improved: WOMAC	**+**			**+**
Knee osteoarthritis (Natalia et al., 2024)	?	KOOS function improved	**+**		**+**	**+**
Knee osteoarthritis (Nazari et al., 2019)	3	Stiffness in knee reduced: WOMAC stiffness	**+**	**+**		**+**
Patellofemoral pain syndrome (Nouri et al., 2019)	1	Effusion reduced: ultrasound	**+**			**+**
Knee osteoarthritis (Özgözen et al., 2025)	2	HILT+ TENS+ET+hot packs reduced pain, but was not superior to placebo light	**+**			-
Patellofemoral pain syndrome (Ozlu and Atilgan, 2024)	1	Flexion ROM increased	**+**	**+**		**+**
Knee osteoarthritis (Rizki et al., 2025 the effectiveness of adding high-intensity laser therapy (HILT) to physical exercise in reducing pain, improving muscle strength, and enhancing functional ability in patients with knee osteoarthritis: a randomized controlled trial, 2025)	1	Function improved	**+**			**+**
Knee osteoarthritis (Roheym et al., 2023)	2	Stiffness reduced	**+**			**+**
Knee osteoarthritis (Samaan et al., 2022)	1	Improved: WOMAC	**+**	**+**		**+**
Knee osteoarthritis (Sen et al., 2024)	2	Function improved	**+**	**+**		**+**
Knee osteoarthritis (Sharma et al., 2025)	?	Function improved: SF-36	**+**			**+**
Knee osteoarthritis (Siriratna et al., 2022)	1	Function: WOMAC	**+**			-
Knee osteoarthritis (Taheri et al., 2023)	1	Function: WOMAC	**+**	**+**		**+**
Knee osteoarthritis (Tangsriwong et al., 2025)	2	Single session, slight increase in muscle strength	**+**	**+**		
Knee osteoarthritis (Wibisono et al., 2024)	U	Improved balance more than LLT				**+**
Spine/back						
Chronic discogenic sciatica (Abdelmageed et al., 2022)	1	↑ Walking distance ↑ neuron activity Conduction recovery	**+**	**+**		**+**
Myofascial trigger points (Ahmed et al., 2020)	1	Pain reduced	**+**			
Myofascial pain syndrome (Ahi and Sirzai, 2022)	1	↓Disability: NDI QoL: SF-36	**+**	**+**	**+**	**+**
Peripheral neuropathy (Chatterjee et al., 2019)	1	↓IL-6 ↓MCP1	**+**		**+**	**+**
Lumbar disc protrusion (Chen et al., 2018)	1	↓Disability: ODI	**+**			-
Hemiparetic osteoparetic stroke (Abo Elyazed et al., 2023)	?	QoL improved: QUALEFFO-41 Balance improved	**+**		**+**	**+**
Myofascial pain syndrome post-neck discectomy (Gawaan et al., 2023)	1	Pressure pain threshold increased neck ROM increased	**+**	**+**		
Neck pain (İnce et al., 2024)	1	↑Cervical ROM ↓disability: NDI QoL: SF-36	**+**	**+**	**+**	**+**
Chronic neck pain (Kenareh et al., 2021)	U	Short term pain relief	**+**			**+**
Fibromyalgia (Kelini et al., 2025)	1	QoL: SF-36	**+**		**+**	
Lumbar disc herniation (Kumar et al., 2025)	2	↑ Lumbar canal diameter	**+**			
Coccydynia (Kumar and Mehta, 2025)	U	HILT was superior to US	**+**			
Myofascial trigger points (Rahbar et al., 2025)	1	Function: ODI	**+**			**+**
Cervical radiculopathy (Sherif et al., 2023)	U	Electrophysiology unchanged	**+**			
Chronic neck pain (Shreestha and Sharma, 2024)	1	↓Disability: NDI	**+**			
Lumbar disc herniation (Song et al., 2018)	1	QoL: SF-36 function: ODI & SF-36	**+**		**+**	**+**
Cervical spondylosis (Venosa et al., 2019)	1	↓Disability: NDI cervical ROM	**+**	**+**	**+**	**+**
Neck pain (Xie et al., 2025)	?	ROM increased Function: NDI	**+**	**+**		-
Upper trapezius myofascial pain syndrome (Yassin et al., 2024)	1	↓Disability: NDI cervical ROM	**+**	**+**		**+**
Cervical disc herniation (Yilmaz et al., 2020)	1	Function and disability: NPADS	**+**	**+**	**+**	
Shoulder/arm						
Hemiplegic shoulder dysfunction/pain (Abdelhakiem et al., 2024)	?	Function: UCLA ↑ handgrip strength	**+**			**+**
Subacromial impingement (Aceituno-Gómez et al., 2019)	2	Disability: CMS score unchanged	**+**			-
Subacromial impingement syndrome (Akbaş et al., 2025)	2	Function: SPADI	**+**			**+**
Adhesive capsulitis (Atan and Bahar-Ozdemir, 2021)	2	Function: SPADI unchanged	**+**		**+**	-
Lateral epicondylitis (Bilir et al., 2024)	2	Function: QuickDASH ↑ Hand grip strength	**+**		**+**	**+**
Hemiplegic shoulder pain (Dajpratham et al., 2024)	2	Pain reduced	**+**	**+**		
Diabetic frozen shoulder (Elerian et al., 2024)	1	Function: ASES	**+**		**+**	**+**
Rotator cuff tendinopathy (Elsodany et al., 2018)	1	Function: SPADI ↑ ROM at EOT & 3 mos	**+**	**+**		**+**
Chronic stroke (Indolia and Alam, 2025)	0	Function: FMA-UE	**+**	**+**		**+**
Lateral epicondylitis (Karaca et al., 2022)	0	↑ Grip strength function: DHI	**+**			**+**
Lateral epicondylitis (Kaydok et al., 2020)	2	↑ Grip strength QoL: SF-36 function: QDASH	**+**		**+**	**+**
Hemiplegic shoulder pain (Korkmaz et al., 2022)	2	↓Disability: SPADI motor recovery improved function: FIM rotator cuff tear size reduced	**+**	**+**	**+**	**+**
Adhesive capsulitis (Ordahan et al., 2023)	2	↓Disability: SPADI	**+**	-		**+**
Subacromial impingement syndrome (Saleh et al., 2025)	2	↑ Sleep quality: PSQI	**+**		**+**	**+**
Lateral epicondylitis (Sen et al., 2024)	0	↓Disability: QDASH	**+**			**+**
Supraspinatus tendon tears (Uzun et al., 2025)	1	Function: SPADI tendon thickness unchanged	**+**		**+**	**+**
Adhesive capsulitis (Uysal et al., 2023)	1	Function: SPADI ROM increased	**+**	**+**		**+**
Subacromial impingement syndrome (Yılmaz et al., 2021)	2	Function: CMS QoL: SF-36 strength increased	**+**	**+**	**+**	**+**
Subacromial impingement syndrome (Zaki et al., 2022)	1	Function: SPADI and MSKUS	**+**			**+**
Bell’s Palsy (Abdullatif et al., 2022)	1	Increased eyebrow raising increased smiling ability		**+**		**+**
Myogenic temporomandibular joint disorder (Ekici et al., 2021)	2	↑ Mouth opening 27% vs. 4% ↓disability: JFLS-20 QoL and function: OHIP-14	**+**	**+**		**+**
Temporomandibular joint disc displacement (Ekici et al., 2022)	1	Mouth opening wider Function: JFLS-20 QoL & Function: OHIP-14	**+**	**+**	**+**	**+**
Chronic sinusitis (Elkalla et al., 2020)	1	Symptomatic improvements: SNOT-22				
Temporomandibular joint disorder (Nadershah et al., 2020)	2	Pain reduced	**+**			
Myogenic temporomandibular disorder (Qataya et al., 2025)	1	QoL and Function: OHIP-14 ↑ mouth opening	**+**	**+**	**+**	**+**
Bell’s Palsy (Wu et al., 2023)	0	Improved symmetry, voluntary movement, and electrophysiology, QoL: FaCE function: HB and SFGS		**+**	**+**	**+**
Hand/wrist						
Hand arthritis in systemic lupus erythematosus (Abdel-Aal et al., 2020)	2	Swelling reduced Tenderness reduced ↑ Grip strength	**+**			**+**
De Quervain’s tenosynovitis (Dundar Ahi and Sirzai, 2023)	2	↑ Hand grip strength QoL: SF-36 function: QDASH	**+**			**+**
Carpal tunnel syndrome (Ashour et al., 2022)	1	Reduction in pain	**+**			
Thumb osteoarthritis (Cantero-Téllez et al., 2019)	3	↑ Pinch strength	**+**			
De Quervain’s tenosynovitis (Chongkriengkrai et al., 2023)	2	Hand grip strength unchanged ↓disability several AEs	**+**			-
Carpal tunnel syndrome (Hojjati et al., 2020)	1	Increased pinch strength	**+**			**+**
Chronic regional pain syndrome thumb osteoarthritis (Khoramdel et al., 2025)	1	↓Effusion ↓swelling ↓function: FMA ↑ nerve function: EMG	**+**	**+**		**+**
Carpal tunnel syndrome (Ezzati et al., 2019)	2	Function: CMAP improved neuromuscular transmission	**+**			**+**
Foot						
Plantar fasciitis (Zare Bidoki et al., 2024)	2	HTI Function and QoL: SF-36	**+**		**+**	**+**
Diabetic peripheral neuropathy (Chatterjee et al., 2019)	1	Reduced pain QoL: SF-36	**+**		**+**	
Hemophilic A ankle arthropathy (Elnaggar, 2020a)	1	Gait improved	**+**			**+**
Calcaneal Spur (Karakuzu Güngör, 2025)	1	Function: FFI	**+**			**+**
Plantar fasciitis (Jitpimolmard et al., 2026)	2	Function and QoL: FAAM	**+**		**+**	**+**
Plantar fasciitis (Ketz et al., 2024)	0	Pain reduced function: FAAM	**+**			**+**
Plantar fasciitis (Kosehasanogullari et al., 2026)	?	Pain reduced function: FFI	**+**			**+**
Plantar fasciitis (Naruseviciute and Kubilius, 2020)	1	Pain reduced	**+**			
Plantar fasciitis (Ordahan et al., 2018)	2	Function: HTI QoL: FAOS	**+**		**+**	**+**
Plantar fasciitis (Thammajaree et al., 2023)	2	Function: FFI	**+**			**+**
Calcaneal spur and plantar fasciitis (Tkocz et al., 2021)	?	No change in pain	-			
Plantar fasciitis (Yadav et al., 2026)	2	Pain reduced function: FFI	**+**			**+**
Calcaneal spur (Yesil et al., 2020)	2	Function: FAOS QoL: SF-36 improved pedographic measures	-		-	**+**
Wounds						
Chronic refractory wounds (Lu et al., 2021)	1	Wound healing improved: BWAT pressure ulcer healing improved: PUSH				
Caesarian healing in diabetic women (Thabet et al., 2018)	1	Wound appearance improved	**+**			
Gynecological						
Endometriosis (Thabet and Alshehri, 2018)	1	Adhesions reduced endometriosis reduced QoL: EHP-5	**+**		**+**	
Bone						
Osteoporosis (Alayat M. S. M. et al., 2018)	1	HILT followed by ET improved lumbar bone mineral density 1 y FU HILT alone produced insignificant gains				
Leg						
Proximal hamstring tendinopathy (Verma et al., 2022)	2	Pain reduced isokinetic peak torque unchanged	**+**			
Brain						
Mild cognitive impairment (Han et al., 2025)	1	Transcranial HILT improved working memory Increased working memory Increased neural connectivity: fMRI				
Heart						
Acute coronary syndrome (Jangra et al., 2024)	2	Decreased myocardial injury: troponin 1 No change in LVEF				
Abdomen						
Abdominal obesity (Abdelaal, 2023)	2	HILT reduced weight and forced vital capacity in patients with abdominal 1 obesity				

Studies are categorized by target anatomies for consideration of the relative depth of penetration: knee, spine/back, shoulder/arm, face, hand/wrist, foot, wounds, gynecological, bone, and COVID-19. Single, double, or triple blinded are denoted by numbers 1–3. HILT specific outcomes are described with effective pain reduction, range of motion (ROM) increases, quality of life (QoL) increases, or functional gains after HILT treatment denoted as positive (+) or negative (-) when assessed. ASES, American Shoulder and Elbow Surgeons; BWAT, Bates- Jensen Wound Assessment Tool; CMAP, compound muscle action potential; CMS, Constant-Murley Score; DHI, Duruoz Hand Index; EMG, electromyography; EOT, end of treatment; EHP-5, Endometriosis Health Profile; EroQoL, European Quality of Life; FAAM, Foot and Ankle Ability Measure; FIM, Functional Independence Measure; FMA-UE, Fugl-Meyer Assessment for Upper Extremity; FFI, Foot Function Index; fNIRS, functional near-infrared spectroscopy; HTI, Heel Tenderness Index; JFLS-20, Jaw Functional Limitation Scale 20; LVEF, left ventricular ejection fraction; MSKUS, musculoskeletal ultrasound imaging; NDI, Neck Disability Index; NPADS, neck pain and disability scale; NPRS, Numerical Pain Rating Scale; ODI, Oswestry Disability Index; OHIP-14, Oral Health Impact Profile; PSQI; Pittsburgh Sleep Quality Index; QuickDASH, Quick Disabilities of the Arm, Shoulder, and Hand; SF-36, Short Form 36 QoL survey; SNOT-22, Sino Nasal Outcome Test; SPADI, Shoulder Pain and Disability Index; UCLA, University of California–Los Angeles functional scale; WOMAC, Western Ontario and McMaster Universities Osteoarthritis Index.

**TABLE 2:** Study characteristics and effect sizes associated with laser therapy conditions.

Knee				
**Diagnosiss**	**Laser settings**	**Treatment**	**Study design** **FU**	**Cohen’s *d* effect size**
Juvenile rheumatoid arthritis (El-Shamy et al., 2018)	10.5W 52.5 W/cm^2^ 750J/knee/session	3x/wk 4 wk	ET ± HILT EOT	4.6
Knee osteoarthritis (Nazari et al., 2019)	4W spot size? 30 Hz 2100 J/session	3x/wk 4 wk	ET ± HILT EOT	4.0
Knee osteoarthritis (Tangsriwong et al., 2025)	10.5W pulsed 100 μs 500 J and 1,250 J	1x	HILT vs. sham EOT	3.7
Hemophilic arthropathy (El-Shamy and Abdelaal, 2018)	10.5 W (52.5 W/cm^2^) 120–150 μs 10–30 Hz; 750 J/knee/session	3x/wk 12 wk	ET ± HILT EOT	2.9
Patellofemoral pain syndrome (Ozlu and Atilgan, 2024)	10 W 4 m and 7 W 6 m	5x/wk 2 wk	HILT vs. US-TENS vs. US-IFC EOT	2.9
Knee osteoarthritis (Anupma et al., 2025)	10 W 10 W/cm^2^ 3,000 J	3x/wk 10 wks	HILT vs. sham EOT	2.7
Knee asteoarthritis (Samaan et al., 2022)	12 W (12 W/cm^2^) 25 Hz 300 J/analgesic or 3000 J/biostimulation	2x/wk 2 wk	ET ± HILT EOT	2.4
Knee osteoarthritis (Bettencourt, 2020)	30 W (30 W/cm^2^) 6 and 80 J	Every other day 14x total 2 wks	HILT vs. sham 4 wk	1.9
Knee osteoarthritis (Siriratna et al., 2022)	1 W (0.5 W/cm^2^) 2,000 Hz 7 560 J/session	2–3x/wk 10x total	HILT vs. sham EOT	1.8
Knee osteoarthritis (Koevska et al., 2025)	4W	5x/wk 2 wk	HILT+ET vs. LLLT+ET 1 mo	1.8
Knee osteoarthritis (Natalia et al., 2024)	10 W 2 m 25 hz 25 cm^2^ then 5 W 4 m 25 Hz 25 cm^2^	2x/wk 4 wks	HILT vs. LLLT	1.7
Knee osteoarthritis (Ciplak et al., 2018)	10 W 4 m & 4 W 6 m	5x/wk 2 wk	ET+US vs. HILT EOT	1.4
Knee osteoarthritis (Ahmad et al., 2023)	5W; 5W/cm^2^ 190 J pulsed & 3000 J CW	1x/wk 12 wks	HILT vs. LLLT EOT	1.3
Knee osteoarthritis (Ceyhan and Karaca, 2025)	10.5 W 52.5 W/cm^2^ 1,500 mJ/cm^2^/session	5x/wk 2 wk	HILT vs. SWD EOT	1.2
Knee osteoarthritis (Taheri et al., 2023)	2 W	3x/wk 2 wk	ET ± HILT 3 mos	1.1
Knee osteoarthritis (Akaltun et al., 2020)	12 W (12 W/cm^2^) 25 Hz 300 J/analgesic 3 tx then 3,000 J/biostimulation 7 tx	5X/wk 2 wk	± HILT 6 wks	1.1
Knee osteoarthritis (Taheri et al., 2023)	2 W 500 J	3x/wk 2 wk	ET ± HILT EOT	1.1
Knee osteoarthritis (Mostafa et al., 2022)	10.5 W 52.5 W/cm^2^ 1,500 mJ/cm^2^/session	3x/wk 4 wk	HILT vs. SWT EOT	1.0
Knee osteoarthritis (Ezzati et al., 2024)	1.6 W 300 s	5x/wk 2 wk	± HILT vs. LLLT EOT and 1 mo	0.7
Knee osteoarthritis (Sen et al., 2024)	?W 300 J 5 tx then 3,000 J 5 tx	5tx/wk 2 wks	HILT+ET vs. LLLT+ET EOT	0.5
Knee osteoarthritis (Ekici and Ordahan, 2023)	10 W 2 m/cm^2^ 1 wk then 5 W 10 m/25 cm^2^ for 2 wks	3x/wk 3 wk	ET ± HILT EOT	0.4
Knee osteoarthritis (Wibisono et al., 2024)	10 W 2 m—analgesic phase 10 W 4 m—biostimulatory phase	2x/wk 4 wk	HILT vs. LLLT EOT	0.3 (balance)
Knee osteoarthritis (Sharma et al., 2025)	?W 1250 J	3x/wk 3 wk	HILT vs. IGP EOT	0.2
Knee osteoarthritis (Laotammateep et al., 2025)	10 W/cm^2^ 600 J 2 tx then 5 W/cm^2^ 3,000 J 4 tx	2x/wk 3 wks	HILT vs. sham EOT	0.1
Knee osteoarthritis (Özgözen et al., 2025)	9.7 W 2,079 J	5x/wk 2 wk	TENS+ET ± HILT 12 wks	0.0
Knee osteoarthritis (Astri et al., 2022)	10 W/cm^2^ 2 m and 5 W/cm^2^ 2 m	3x/wk 2 wks	HILT+ET vs. LLLT+ET EOT	↓Pain
Knee osteoarthritis (Rizki et al., 2025)	10 W 300 J then 5 W 300 J	?	ET+HILT/sham	↓Pain
Patellofemoral Pain Syndrome (Nouri et al., 2019)	10W	5x/wk 2 wk	HILT vs. sham EOT	Balance Improved
Spine/back				
Lumbar disc herniation (Song et al., 2025)	15 W max, 890/910 nm power density unknown 900 J/min, 4–6 min/session	5x days	Collagenase ± HILT 1 wk	3.3
Cervical spondylosis (Venosa et al., 2019)	10.5 W 52.5 W/cm^2^ 120–150 μs, 10–30 Hz 2050 J/session	2x/wk 6 wks	HILT+ET vs. US+TENS+ET 4 wks	3.3
Myofascial trigger points (Ahmed et al., 2020)	3 watts 810 and 980 nm	2x/wk 4 wk	HILT+ET vs. TENS+ET	2.5
Cervical radiculopathy (Sherif et al., 2023)	2 W & 4 W 980 nm 4 Hz 840 J/session	2x/wk 4 wks	ET ± HILT EOT	2.3
Fibromyalgia (Kelini et al., 2025)	10.5 W 140 J	3x/wk 6 wks	ET ± HILT EOT	1.9
Neck pain (İnce et al., 2024)	10.5 W 52.5 W/cm^2^ 120–150 μs 10–30 Hz 2,632 J/session	5x/wk 4 wks	ET ± HILT 12 wks	1.8
Myofascial trigger points (Rahbar et al., 2025)	15 W 30 kHz 20–50% duty 810 and 980 nm	5x/wk 2 wk	HILT vs. PT EOT	1.7
Hemiparetic osteoparetic stroke (Abo Elyazed et al., 2023)	10.5 W 3,000 J 10 m per spot	3x/wk 12 wks	HILT vs. SoC EOT	1.6
Chronic lower back pain (Abdelbasset et al., 2020)	12W 12 W/cm^2^ 1,200 J/session	2/wk 12 wks	HILT vs. sham EOT	1.5
Lumbar disc herniation (Kumar et al., 2025)	9 W 3,780 J 980/810 nm	3x/wk 10 wks	HILT vs. sham EOT	1.6
Myofascial pain syndrome post-neck discectomy (Gawaan et al., 2023)	14 W 2,803 J/tx	3x/wk 4 wk	ET ± HILT EOT	1.4
Chronic discogenic sciatica (Abdelmageed et al., 2022)	1 W/cm2 810 nm + 980 nm 400 J/session	3x/wk 4 wks	PT ± HILT EOT	1.4
Chronic neck pain (Kenareh et al., 2021)	10 W 3 m then 7 W 7 m	1/d 2 wks 10x total	HILT vs. US EOT	1.4
Myofascial pain syndrome post-neck discectomy (Gawaan et al., 2023)	14 W 2,803 J	3x/wk 1 mo	ET ± HILT EOT	1.4
Neck pain (Xie et al., 2025)	8 W 2,800 J	One treatment	HILT vs. sham EOT	1.3
Lumbar disc protrusion (Chen et al., 2018)	12 W 12 W/cm^2^ 7,500 J/session	5x/wk 2 wk	± HILT+Spinal decompression 4 wks	1.0
Peripheral neuropathy, lumbar (Chatterjee et al., 2019)	8 W 5.3 W/cm^2^ 980 nm:810 nm at 80%:20% 1920 J	3x/wk 4 wks 1x/wk 8wks	HILT+SoC vs. Sham+SoC EOT	0.9
Cervical disc herniation (Yilmaz et al., 2020)	8 W 8 W/cm^2^ 1,850 J/session	5x/wk 4 wk	ET ± HILT EOT	0.3
Upper trapezius myofascial pain syndrome (Yassin et al., 2024)	6.5 W 2,728 J	2x/wk 3 wks	HILT+ET vs. DN+ET EOT	-0.4
Coccydynia (Kumar and Mehta, 2025)	6 W 1,000 J HIRO 1.0	12 tx/2 wks	HILT+US vs. US EOT	↓Pain
Shrestha chronic neck pain	10 W 810 and 980 nm	3x/wk 2 wks	HILT vs. US	↓Pain
Myofascial pain syndrome (Ahi and Sirzai, 2022)	8 W 8 W/cm^2^	2x/wk 4 wk	ET+PT ± HILT EOT	Don’t have raw data, but (+)
Shoulder/arm				
Subacromial impingement syndrome (Saleh et al., 2025)	4 W, 5 W/cm^2^ 300 s	3x/wk 3 wks	ET ± HILT EOT	6.0
Lateral epicondylitis (Karaca et al., 2022)	4 and 6 W 13–20 W/cm^2^ 6 J/cm2 analgesic and 12 J/cm2 biostimulation	2x/wk 2 wks	PT+ESWT ± HILT 4 wks	5.0
Rotator cuff tendinopathy (Elsodany et al., 2018)	Power? 10.5 W? 2,050 J per session	3x/wk 4 wks	ET ± HILT EOT	4.7
Hemiplegic shoulder pain after stroke (Abdelhakiem et al., 2024)	3x 8 W 75 s 100 J 6x 12 W 30 s	3x/wk 3 wks	ET ± HILT 1 wk	4.5
Partial supraspinatus tendon tears (Uzun et al., 2025)	8 and 7 W 8 w/cm^2^ 2,500 J	3x/wk 3 wk	HILT vs. US 4 wk	2.8
Adhesive capsulitis (Atan and Bahar-Ozdemir, 2021)	8, 12, 8 W 100 J/cm^2^	5x/wk 3 wk	ET ± HILT 3 mos	2.2
Adhesive capsulitis (Ordahan et al., 2023)	12 W; 12 W/cm^2^ 10 J/cm^2^/analgesic and 100 J/cm^2^/biostimulatory	5 x/wk 3 wk	HILT ± stretchin+ET Vs. LLLT ± stretchin+ET 3 wks	1.6
Subacromial impingement syndrome (Akbaş et al., 2025)	10 W 100 s first 5 tx 250 J 4 W 10 m next 5 tx 2,500 J	5x/wks 2 wk	HILT+ET vs. sham+ET EOT	1.4
Hemiplegic shoulder pain (Korkmaz et al., 2022)	8 W; 8 W/cm^2^ 300 J/analgesic session and 2,500 J/biostimulatory session	3x/wk 3 wk	PT ± HILT EOT	1.3
Diabetic frozen shoulder (Elerian et al., 2024)	10 W 52.5 W/cm^2^ 1,080 J	2x/wk 8 wk	HILT vs. SWT EOT	1.1
Diabetic peripheral neuropathy (Chatterjee et al., 2019)	4–10 W for 360–1,800 J Focused on heel and lumbar regions	2x/wk 4 wks, then 1x/wk 8 wks	HILT vs. sham EOT	0.9
Elbow tendinopathy (Yüksel et al., 2025)	8 W	3x/wk 3 wks	HILT vs. ET EOT	0.7
Subacromial impingement syndrome (Yilmaz et al., 2020)	10.5 W; 52.5 W/cm^2^ 40 Hz; 2,781 J/session	5x/wk 3 wks	ET ± HILT EOT	0.4
Lateral epicondylitis (Kaydok et al., 2020)	6 W; 6 W/cm^2^ 6 J/cm^2^/analgesic and 120–150 J/cm^2^/biostimulatory	3x/wk 3 wk	HILT vs. LLLT EOT	0.4
Subacromial impingement (Aceituno-Gómez et al., 2019)	12 W pulsed 250 J/cm^2^	5x/wk 3 wk	ET ± HILT 3 mos	0.3
Lateral epicondylitis (Bilir et al., 2024)	4 W 300 J 3x 6 W 12.5 m 4,500 J 6x	3x/wk 3 wk	HILT vs. ESWT	↓Pain
Hemiplegic shoulder pain (Dajpratham et al., 2024)	10 W 52.5 W/cm^2^ 2,500 J	5x/wk 2 wk	HILT+ET vs. US+ET	↓Pain
Subacromial impingement syndrome (Zaki et al., 2022)	4 W; 5 W/cm^2^ 2,050 J/session	3x/wk 7 sessions total	LLLT-KT, HPL-KT, and sham-KT 2 d	↓Pain
Lateral epicondylitis (Sen et al., 2024)	15 W	5x/wk 3 wks	HILT+SoC vs. ESWT+SoC 3rd and 12th week	↓Pain
Adhesive capsulitis (Uysal et al., 2023)	8 E 2.5 m and 7 W 6 m 2,500 J	3x/wk 3 wk	HotPacks+TENS+US ± HILT	↓Pain
Face				
Myogenic temporomandibular joint disorder (Ekici et al., 2021)	10.5 W 52.5 W/cm^2^ 120–150 μs 10–30 Hz 1029 J/session	5x/wk 3 wks	HILT vs. sham 4 wk	1.5
Temporomandibular joint disc displacement (Ekici et al., 2022)	10.5 W 52.5 W/cm^2^ 1,060 J	5x/wk 3 wk	HILT vs. control vs. TENS 1wk	1.6
Bell’s Palsy (Wu et al., 2023)	1 W of 808 nm + 0.2 W of 905 nm	3x/wk 72x total	± HILT EOT	1.2
Temporomandibular joint disorder (Nadershah et al., 2020)	7 W 2.5 W/cm^2^ 300J/treatment	Every other day 10 d	HILT vs. sham EOT	↓Pain
Bell’s Palsy (Abdullatif et al., 2022)	1,808 J/session	1x/wk 4 wk	2 sides of face EOT	↑ Function
Chronic sinusitis (Elkalla et al., 2020)	1 W/cm^2^ 810 nm and 980 nm 150 J/sinus	3x/wk 4 wk	SMT ± HILT EOT	↑ Function
Myogenic temporomandibular disorder (Qataya et al., 2025)	2 W, 200 mw/cm^2^, 10 hz, 1 m/spot then 5 W, 550 mW/cm^2^, 60 Hz 2 m/spot	1x	HILT vs. imEGF	↓Pain ↑ mouth opening QoL improved
Hand/wrist				
Chronic regional pain syndrome (Khoramdel et al., 2025)	5 W 660, 800, 905, and 970 nm	6x/2wks	HLIT vs. sham 2 d	4.1
Hand arthritis in SLE (Abdel-Aal et al., 2020)	Power? 2,100 J/session	3x/wk 8 wks	HILT vs. sham EOT	1.9
De Quervain’s tenosynovitis (Ahi and Sirzai, 2022)	10 W 7x and 6 W 8x 250 J	3x/wk 5 wks	HILT vs. placebo laser EOT	1.0
Thumb osteoarthritis (Cantero-Téllez et al., 2019)	1.5 W 0.3 W/cm^2^ 800 and 970 nm 2 Hz 50% duty cycle 75 J/session	3x/wk 4 wks	HILT vs. sham EOT	1.2
De Quervain’s tenosynovitis (Dundar Ahi and Sirzai, 2023)	12 W 52.5 W/cm^2^ 10 J/cm^2^/analgesic and 120–150 J/cm^2^/biostimulatory	3x/wk 5 wk	HILT vs. sham EOT	1.0
Carpal tunnel syndrome (Ezzati et al., 2020a) Iran; 31742366	1.6 W 808 nm 8 J or 20 J/cm^2^	5x/2 wks	HILT vs. LLLT EOT	0.4
De Quervain’s tenosynovitis (Chongkriengkrai et al., 2023)	8 W 8 W/cm^2^ 80 J/analgesic and 800 J/biostimulatory	3x/wk 3 wk	Splint+ET+HILT/sham 3 wk	-0.9
Carpal tunnel syndrome (Hojjati et al., 2020)	5 W 180 J	1x	HILT vs. LLLT vs. splint EOT	Could not calculate
Foot				
Plantar fasciitis (Ordahan et al., 2018)	8 and 6 W First week: 8 W 6 J/cm^2^ Next 2 wks: 6 W 120 J/cm^2^	3x/wk 3 wks	HILT vs. LLLT EOT	2.2
Plantar fasciitis (Ketz et al., 2024)	10 W or 25 W 20% 810 nm and 80% 980 nm 10J/cm^2^	3x/wk 3 wks	SoC ± HILT 6 wks	1.5
Diabetic peripheral neuropathy (Chatterjee et al., 2019)	2–10 W at 0.8–4.0 W/cm^2^ 980 nm : 810 nm at 80% : 20% 360–1,800J	3x/wk 4 wks	HILT vs. sham EOT	0.9
Calcaneal spur (Karakuzu Güngör, 2025)	10 W then 7 W	5x/wk 2 wks	ESWT vs. HILT EOT	0.9
hemophilic an ankle arthropathy (Elnaggar, 2020a)	7 W 7 W/cm^2^ 3,000 J	3x/wk 8x total	PT ± HILT EOT	0.7
Plantar fasciitis (Naruseviciute and Kubilius, 2020)	10.5 W 52.5 W/cm^2^ 120–150 μs 10–30 Hz 3,000 J	8x/3wk	PT ± HILT EOT	0.5
Plantar fasciitis (Jitpimolmard et al., 2026)	10 W 10 W/cm^2^ 3,000 J	3x/wk 3 wks	HILT+ET vs. sham+ET EOT	0.5
Plantar fasciitis (Kosehasanogullari et al., 2026)	7 W 4,496 J	5x/wk 3 wks	HILT vs. ESWT vs. ET EOT	0.3
Plantar fasciitis (Thammajaree et al., 2023)	6 W 30 W/cm^2^ 150 J 5 m total	2x/wk 3 wks	HILT vs. ESWT 1 wk	0.2
Plantar fasciitis (Zare Bidoki et al., 2024)	30 W 3 W/cm^2^ 8 J/cm^2^	3x/wk 3 wks	ESWT vs. HILT 3 mos	0.2 (Both effective)
Calcaneal spur (Yesil et al., 2020)	10.5 W 52.5 W/cm^2^ 120–150 μs 10–30 Hz 1281 J/session	5x/wk 3 wks	ET+HILT/sham 4 wks	0.1
Wounds				
Chronic refractory wounds (Lu et al., 2021)	8 W 8 W/cm^2^ 80 J/cm^2^	3x/wk 3 wk	SMT+HILT/sham EOT	4.2
Caesarian healing in diabetic women (Thabet et al., 2018)	10.5 W 52.5 W/cm^2^ 120–150 μs 10–40 Hz 360–540J	3x/wk 8 wk	SMT+HILT/sham EOT	0.9
COVID-19				
COVID-19 (Vetrici et al., 2021)	500 mW 808 nm 75 mW/cm^2^ 203 mW 905 nm 31 mW/cm^2^ 3,590 J	1x/d 4 d	SoC ± HILT 5 month	No sequalae for HILT group; 40% long-term sequelae control group
COVID-19 ageusia (Shabaan et al., 2023)	1 W 6 m 904 nm	1x	HILT vs. sham 1, 2, 3, and 4 wks	Higher restoration of taste ability
GYNECOLOGICAL				
Endometriosis (Thabet and Alshehri, 2018)	10.5 W 52.5 W/cm^2^ 120–150 μs 10–40 Hz 0.1% duty cycle 1,300 J	3x/wk 8wk	HT ± HILT EOT	2.2
BONE				
Osteoporosis (Alayat M. S. M. et al., 2018)	10.5 W 52.5 W/cm^2^ 120–150 μs 10–30 Hz 3,000 J	3x/wk 24 wks	HILT vs. ET vs. sham vs. HILT+ET EOT	0.5
Abdomen				
Obesity (Abdelaal, 2023)	10.5 W	2x/wk 12 wks	AET ± HILT 3 d	Wt reduced BMI reduced WC reduced FVC increased FEV1 increased
Heart				
Acute coronary syndrome (Jangra et al., 2024) India	6 W 60 s to each of 3 locations	1x/d 3 d	HILT vs. sham 1, 2, 3 d	Troponin 1 reduced
Leg				
Proximal hamstring tendinopathy (Verma et al., 2022)	5 W 980/810 1,800 J	3x/wk 3 wks	HILT vs. CT	0.3
Brain				
Mild cognitive impairment (Han et al., 2025)	3.4 W, 1,064 nm 250 mW/cm^2^ 3,672 J	1 tx	TMS+HILT vs. sham+HILT vs. TMS+sham vs. sham both were EOT	Working memory improved DPFC improved hemodynamics (fMRI)

1,064 nm wavelengths were used throughout unless noted otherwise under treatment settings. FU-follow-up; ET-exercise therapy; PT-physical therapy; ESWT-extracorporeal shock wave therapy; KT-kinesiology taping; LLL-low-level light; HT-hormone therapy; SMT-standard medical treatment; SWD-short-wave diathermy; TMS-transcranial magnetic stimulation; EOT-end of treatment follow-up; UC, usual care; NC, not calculated.. Kaydock study was a measure of hand grip strength and Abdel-all was a measure of joint swelling; AET-aerobic exercise therapy. Wt - weight, WC - waste circumference, FVC - forced vital capacity, FEV1 - forced expiratory volume in 1 s, DLPFC - left dorsolateral prefrontal cortex. All other measures were VAS. Studies are summarized by laser parameters, study design, treatment plans, follow-up, and respective Cohen’s *d* effect size when it could be calculated from published data. Cohen’s *d* represents the standardized mean difference between the laser treatment and control groups, expressed in pooled standard deviation units. Effect sizes were directionally coded so that positive values favor laser treatment and negative values favor control; outcomes in which lower scores indicate improvement were reverse-coded so that higher effect sizes consistently indicate better outcomes with laser treatment. BMI, body mass index; CT, conventional therapy; DN, dry needling; FVC, forced vital capacity; imEGF, intra-muscular epidermal growth factor; KT, kinesiology taping; Wt, weight; WC, waste circumference; ESWT, extracorporeal shock wave therapy; FEV1, forced expiratory volume in 1 s; PT, physical therapy; TENS, transcutaneous electrical stimulation;

### 3.2 Clinical outcomes

#### 3.2.1 Global pain meta-analysis—high-intensity laser therapy produces large and sustained reductions in pain

Pain was the most studied outcome metric. HILT treatment reduced pain in 69 out of 75 studies that measured pain using a significance threshold of *p* < 0.05. The effect sizes were large (Cohen’s *d* > 0.8) in 52 out of 69 studies for which effect sizes could be calculated. The standardized mean difference (SMD; Cohen’s *d*) for all pain end of treatment measures was -1.09 (95% CI, -0.84 to -1.33). Most measures (*k* = 56) used the visual analog scale (VAS), but the Shoulder Pain and Disability Index (SPADI), Numerical Pain Rating Scale (NPRS), or the Western Ontario MacMaster (WOMAC) scales were also used (*k* = 6). Consideration of these additional pain measures yielded similar pooled effect estimates (unpublished observation).

Durable analgesic effects were evident, with HILT producing large reductions in VAS pain scores at the end of treatment and sustaining similarly large effects across all follow-up periods beyond treatment cessation (Figure 2). At the end of treatment (50 studies), the standardized mean difference (SMD) was -0.98; this effect was maintained and slightly increased at < 3-month follow-up (19 studies; SMD -1.18) and remained large at ≥ 3-month follow-up (21 studies; SMD -1.01). The overlapping 95% confidence intervals indicate no meaningful attenuation of effect over time. Consistent with this, sustained benefits observed in extended follow-up after treatment cessation, indicated that pain relief is enduring in most cases rather than transient.

**FIGURE 2:**
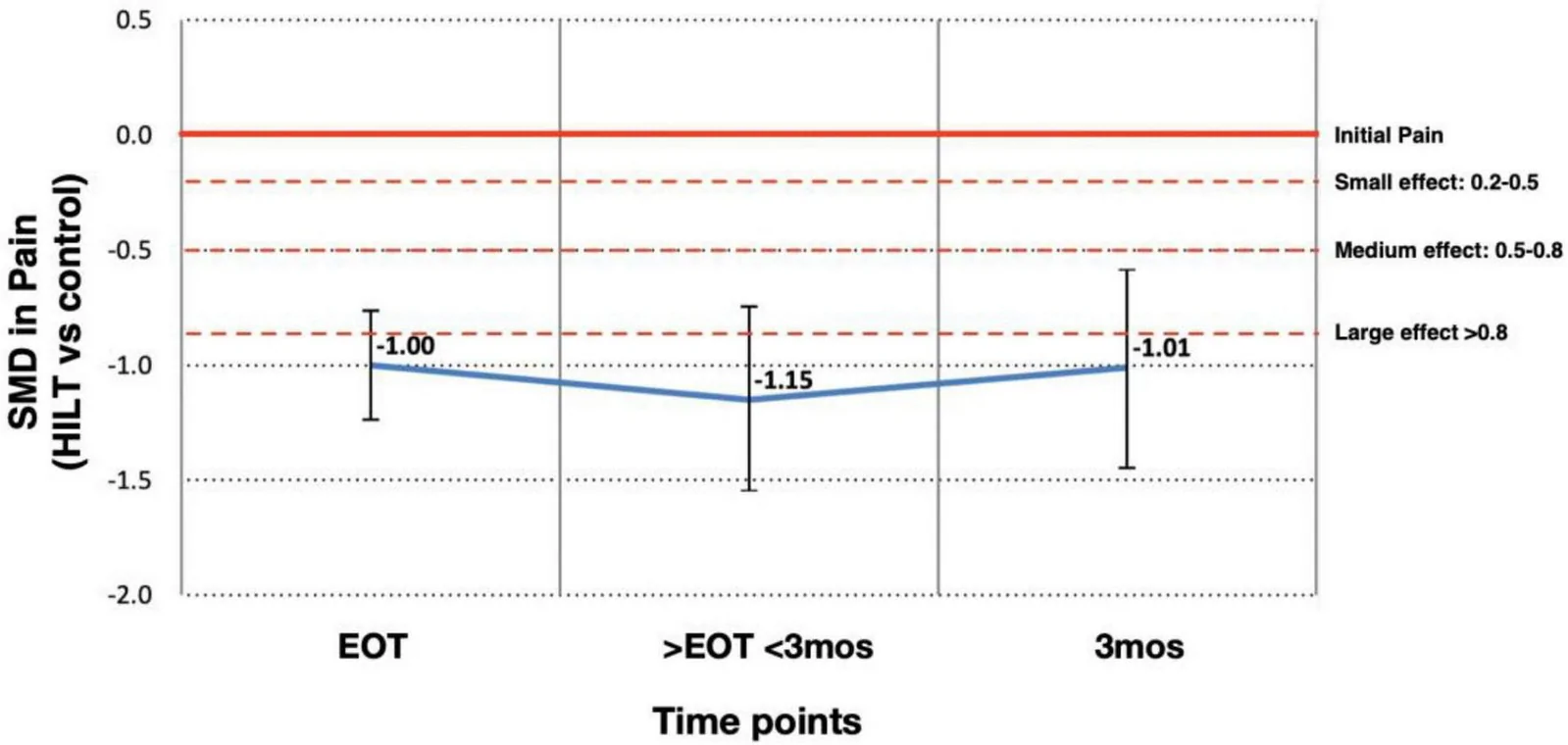
Pooled standardized mean differences (SMDs) in VAS pain scores at end of treatment (EOT), > EOT but < 3-month follow-up, and ≥ 3-month follow-up. Values below 0 favor HILT for pain reduction. Each point represents the pooled SMD in pain reduction for studies reporting VAS outcomes at that time point (EOT: *k* = 53; > EOT < 3 months: *k* = 19; ≥ 3 months: *k* = 21), with vertical whiskers indicating the corresponding 95% confidence intervals.

#### 3.2.2 Pain by anatomical category meta-analysis with consideration of the respective need for power or treatment plan frequency

We compared pain effect sizes across laser power settings and treatment plans to begin identifying optimal parameters for specific anatomical diagnoses. Analyses focused on knee, shoulder/arm, and spine conditions, which provided the largest datasets, and forest plots were generated for these categories as follows (Tables 2, 3). Power densities were considered where the spot sizes were reported.

**TABLE 3:** Evidence-informed optimal high-intensity laser photobiomodulation parameters and treatment regimens by anatomical region with general consideration of pathology depth.

Anatomy	Power density	“Dose” (as Joules or Fluence, J/cm^2^)	Optimal treatment plan
Knee (arthropathies, injuries)	12–52.5 W/cm^2^	750–3,000 J	Daily treatment regimens for at least 2 weeks
Spine/back (radiculopathies, spondylosis, neck pain, chronic pain, myofascial pain syndrome post-neck discectomy, discogenic sciatica, lumbar disc protrusion)	12–52.5 W/cm^2^	850–7,500 J	Daily treatment regimens for at least 2 weeks
Shoulder/arm (lateral epicondylitis, rotator cuff tendinopathy, adhesive capsulitis, frozen shoulder, subacromial impingement syndrome)	5–20 W/cm^2^	2,000–2,500 J	3x/wk for ≥ 2 wks
Wound (chronic refractory wounds)	8–10 W/cm^2^	80 J + 150 J/cm^2^ or 540 J	3x/wk for ≥ 3 wks
Hand/wrist (arthropathies, carpal tunnel syndrome, De Quervain’s tenosynovitis)	0.3–12 W/cm^2^	2,100 J or 150 J/cm^2^	3x/wk for ≥ 4 wks
Foot (plantar fasciitis, ankle arthropathy)	8 W/cm^2^	120 J/cm^2^	3x/wk for 3 wks

These recommended ranges are intended as evidence-informed starting points for protocol selection and hypothesis generation and should not be interpreted as definitive dose response optima; parameters may require adjustment based on individual patient characteristics, device specifications, and concurrent therapies.

##### 3.2.2.1 Knee—large and dose-dependent pain reduction in knee osteoarthritis

More studies focused on *knee* osteoarthritis than any other diagnosis. Pain was consistently reduced in 27 out of 27 studies (Table 2 and Supplementary Table 1) with 18 out of 25 exhibiting a large effect size in pain reductions. Forest plot analysis of 21 studies limited to end-of-treatment outcome measures yielded a SMD of -1.26 with a 95% confidence interval of -1.70 to -0.81 and a significance, *p* < 0.05 (Figure 3). All told studies involved a total of 324 subjects in the HILT cohort and 323 subjects in the control group with analysis limited to end-of-treatment pain measures. The *I*^2^ value indicated that 90% of the variability among studies arises from heterogeneity rather than random chance. Power settings ranged from 1 W to 30 W at power densities ranging from 10 to 52.5 W/cm^2^. The greatest effect sizes were achieved at the higher power densities, where 3 out of the top 4 effect sizes used a power density of 52.5 W/cm^2^ (ES = 2.9–4.6), and most of the remaining studies used 10–12 W/cm^2^ (ES = 0.2–2.4). An additional study focused on the knee demonstrated improvements in balance in patients with knee osteoarthritis only after treatment with HILT but not when treated with LLLT (Wibisono et al., 2024).

**FIGURE 3:**
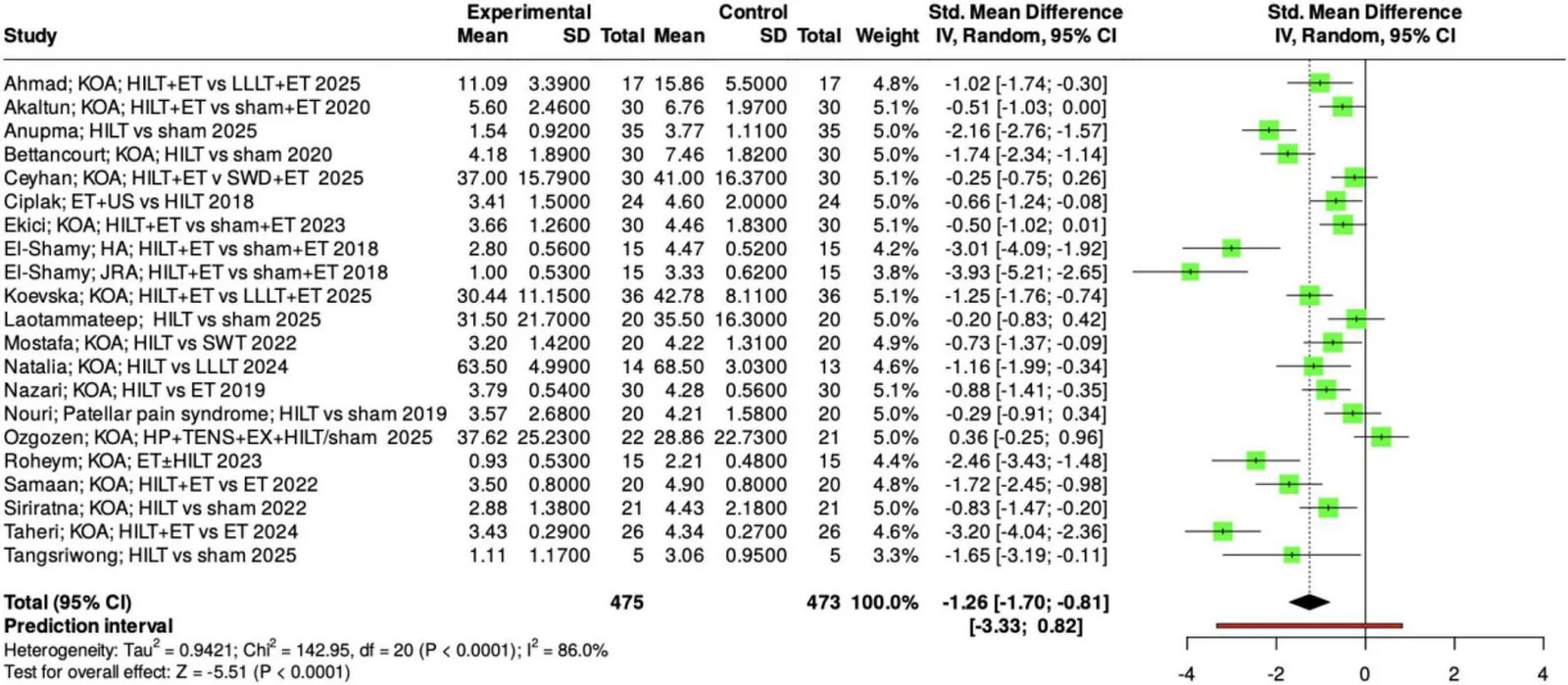
Knee-focused forest plot analysis of outcomes. Analysis was limited to VAS pain measures and end-of-treatment outcomes presented by mean with standard deviation data in the respective studies. KOA, knee osteoarthritis; ET, exercise therapy; US, ultrasound; HA, hemophilic arthropathy; JRA, juvenile rheumatoid arthritis; PPS, patellofemoral pain syndrome; SWT, shock wave therapy; SWD, short-wave diathermy.

##### 3.2.2.2 Shoulder and arm—greatest pain reduction observed in shoulder and arm pathologies

Shoulder and arm pathologies demonstrated the largest therapeutic response to HILT of any anatomical region. All 20 studies targeting shoulder or arm conditions reported pain reduction, with 12 of 14 VAS-based studies (86%) showing large effect sizes (Table 2 and Supplementary Table 1). Meta-analysis of these 14 studies (*n* = 654; 332 HILT, 322 control) revealed a pooled standardized mean difference of -1.55 (95% CI -2.32 to -0.78; *p* < 0.05; Figure 4), the greatest pain reduction across all anatomical categories examined (compare Figures 4–6). Significant therapeutic benefits were observed across diverse shoulder/arm pathologies: subacromial impingement syndrome (*k* = 5), hemiplegic/stroke-related pain (*k* = 4), adhesive capsulitis (*k* = 4), lateral epicondylitis (*k* = 4), rotator cuff tendinopathy, supraspinatus tear, and elbow tendinopathy. Substantial heterogeneity was present (*I*^2^ = 92%).

**FIGURE 6:**
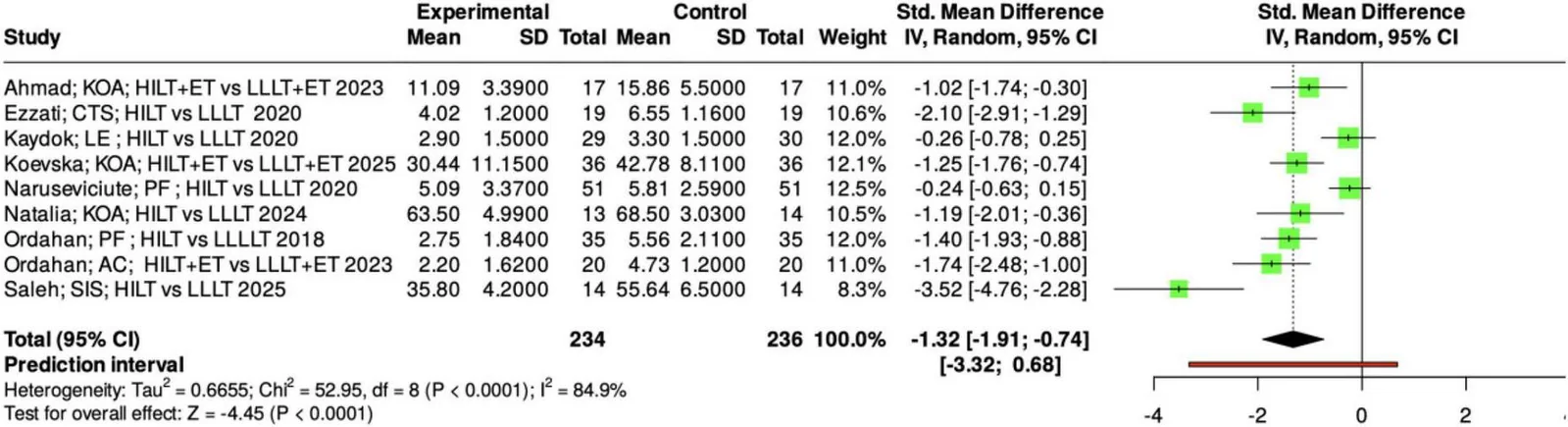
HILT vs. LLLT outcome analysis by forest plot. CTS, carpal tunnel syndrome; PF, plantar fasciitis.

**FIGURE 4:**
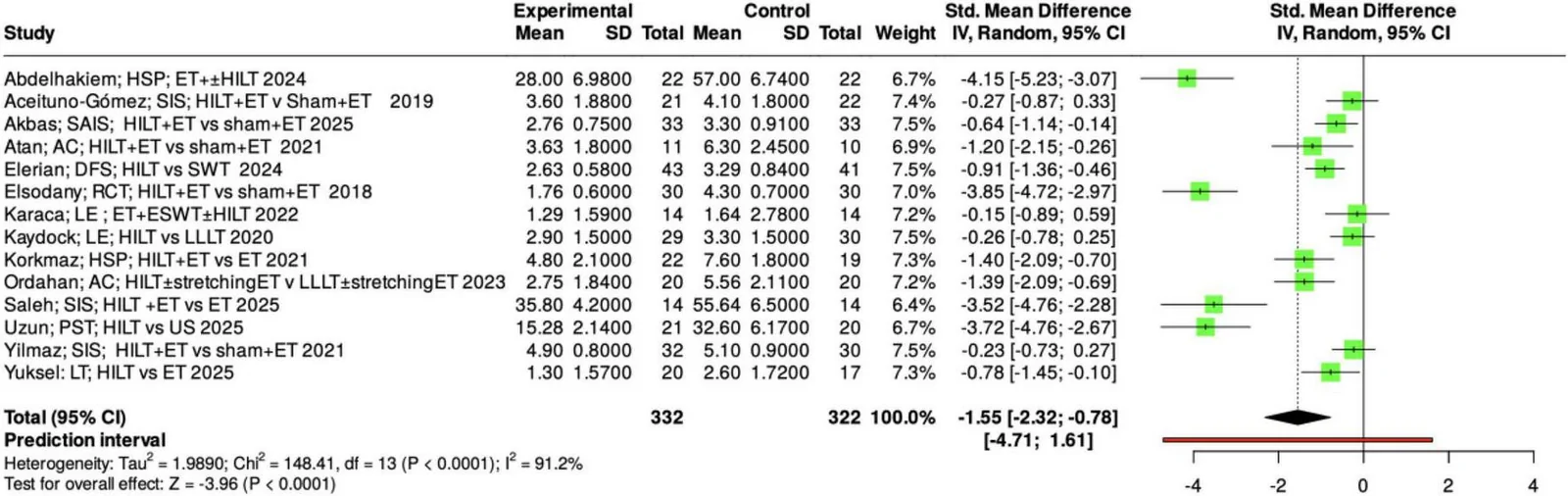
Shoulder/arm-focused forest plot analysis of outcomes. Analysis was limited to VAS pain measures and end-of-treatment outcomes presented by mean with standard deviation data in the respective studies. HSP, hemiplegic shoulder pain/dysfunction; SIS, subacromial impingement syndrome; AC, adhesive capsulitis; DFS, diabetic frozen shoulder; RCT, rotator cuff tendinopathy; LE, lateral epicondylitis; STT, supraspinatus tendon tears; LET, lateral elbow tendinopathy; ESWT, extracorporeal shockwave therapy.

The greatest effect size in this entire meta-analysis was observed for the treatment of subacromial impingement syndrome using 4 W of 810 + 980 nm light as compared to exercise therapy without laser (ES = 6.0; SPADI metric) (Saleh et al., 2025). Most significantly, 4 W yielded superior efficacy compared to LLLT, with the second-largest effect size observed in this entire meta-analysis (ES = 5.3). Another impressive quantitative positive VAS outcome (ES = 5.0) was achieved when using 6 W (1,064 nm, BTL-6000) to treat patients with lateral epicondylitis (Karaca et al., 2022). Treatment involved an initial 45 s of 4 W (12 W/cm^2^) to provide analgesia, followed by 12 min of 6 W administration. Two independent studies focused on post-stroke patients with hemiplegic shoulder pain both yielded large effect size positive outcomes in pain reduction (ES = 4.5 and 1.3) after treating patients with 8 watts 3x per week for 3 weeks (Korkmaz et al., 2022; Abdelhakiem et al., 2024). The results of two studies evaluating HILT treatment for de Quervain’s tenosynovitis were contrasting and interesting (Chongkriengkrai et al., 2023; Dundar Ahi and Sirzai, 2023). A positive outcome (ES = 1.0) with no adverse events was achieved with a higher power and shorter treatment duration, while one patient experienced swelling at the wrist after a longer treatment, which resolved quickly after applying a cold pack (10 W 100 s 250 J vs. 8 W 5 min 800 J, respectively; BTL-6000 1,064 nm for both).

##### 3.2.2.3 Spine/back—HILT effective for all spine and neurological pathologies studied

HILT treatment showed significant pain reduction in *all* 20 *spine or neurological* studies, including myofascial pain syndrome (*k* = 5), cervical neck pain (*k* = 4), lumbar/cervical disc herniation (*k* = 3), cervical radiculopathy (*k* = 2), discogenic sciatica, peripheral diabetic neuropathy, lumbar disc protrusion, post-stroke hemiparesis with osteoporosis, fibromyalgia, coccydynia, and cervical spondylosis. From 13 studies limited to end-of-treatment pain measures, a total of 369 subjects in the HILT cohort and 366 subjects in the control cohort yielded a SMD of -1.22 with a 95% confidence interval of -1.58 to 0.86 and a significance, *p* < 0.05 (Figure 5). A significant heterogeneity was detected (*p* = 0.03), suggesting inconsistent effects in magnitude and/or direction. The *I*^2^ value indicates that 81% of the variability among studies arises from heterogeneity rather than random chance.

**FIGURE 5:**
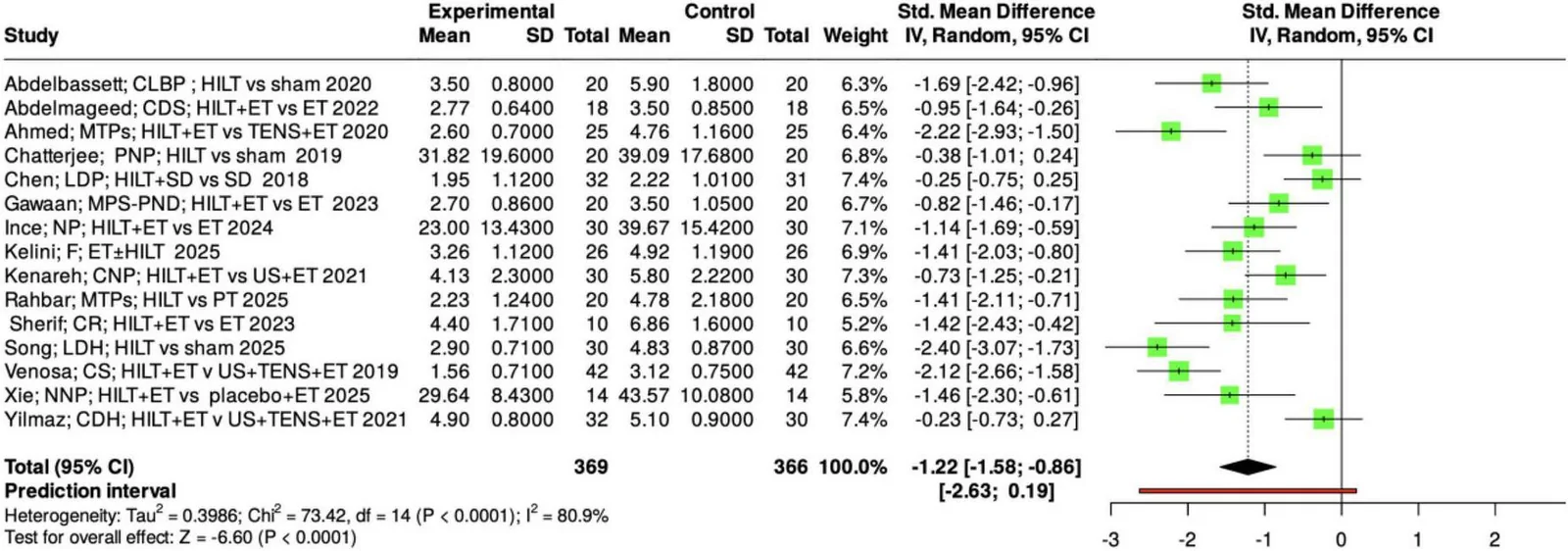
Spine-focused forest plot analysis of outcomes. Analysis was limited to VAS pain measures and end-of-treatment outcomes presented by mean with standard deviation data in the respective studies. CLBP, chronic lower back pain; CDS, chronic discogenic sciatica; MTPs, myofascial trigger points; PNP, peripheral neuropathic pain; LDP, lower disc protrusion; CR, cervical radiculopathy; F, fibromyalgia; CNNP, chronic non-specific neck pain; LDH, lumbar disc herniation; CS, cervical spondylosis; NNP, non-specific neck pain; CDH, cervical disc herniation; SD, spinal decompression; TENS, transcutaneous neurostimulation; MPS, myofascial pain syndrome; MPS-PND, myofascial pain syndrome after post-neck discectomy; CNP, chronic neck pain.

Power settings ranged from 1 to 14 W. The study with the highest effect size (ES = 3.3) involved treatments using 10.5 W (50 W/cm^2^, 1,064 nm, 120–150 μs, 10–40 Hz, 2,050 J total, over 30 min, HIRO 3.0) for patients with cervical spondylosis (Venosa et al., 2019). These treatments were administered twice a week for a duration of 6 weeks.

##### 3.2.2.4 Foot—large effect sizes for plantar fasciitis and ankle disorders

In foot indications, 11 out of 13 HILT treatments resulted in statistically significant pain reduction, specifically for plantar fasciitis (*k* = 9), calcaneal spur (*k* = 3), ankle arthropathy, and peripheral foot neuropathy (Table 1). Five of these studies demonstrated large effect size pain reductions (Ordahan et al., 2018; Chatterjee et al., 2019; Elnaggar, 2020b; Naruseviciute and Kubilius, 2020; Yesil et al., 2020; Zare Bidoki et al., 2024; Karakuzu Güngör, 2025). The most positive outcome (ES = 2.2) was achieved for treatment of plantar fasciitis using 8 W of 1,064 nm light at 8 W/cm^2^ power density to deliver 150 J three times in the first week followed by 6 W power (6 W/cm^2^) to deliver 120 J three times per week for a subsequent 2 weeks (Ordahan et al., 2018). Overall, power densities ranged from 3 to 10 W/cm^2^, with the sole exception of one study using the Hiro 3.0 laser, which used a power density of 52.5 W/cm^2^ to successfully treat ankle arthropathy in children with hemophilia for a large effect size in pain reduction (Elnaggar, 2020b).

##### 3.2.2.5 Hand—consistently positive outcomes for hand and wrist conditions

HILT demonstrated substantial therapeutic efficacy for pain reduction and functional improvement across hand and wrist pain syndromes, including carpal tunnel syndrome (*k* = 3), De Quervain’s tenosynovitis (*k* = 2), thumb osteoarthritis, complex regional pain syndrome (CRPS), and SLE-related hand arthropathy. All eight hand-focused studies reported measurable pain reduction, with large effect sizes in 5 of 7 trials where Cohen’s *d* was calculable. The largest effect size (ES = 4.1) occurred in CRPS using a multi-wavelength protocol (660, 800, 905, 970 nm) at 5 W, delivered 5 × /week for 2 weeks, which improved pain, swelling, function, range of motion, and electromyographic activity (Khoramdel et al., 2025). The second-largest effect, in thumb osteoarthritis, was achieved with dual wavelengths (800 and 970 nm) at 1.5 W (2 Hz, 50% duty) three times weekly for 4 weeks, suggesting that multi-wavelength, moderate-power protocols can yield robust outcomes across diverse hand conditions (Cantero-Téllez et al., 2019).

De Quervain’s tenosynovitis responded favorably in two trials (Chongkriengkrai et al., 2023; Dundar Ahi and Sirzai, 2023). In 64 patients, HILT at 10 W initially and 6 W for biostimulation, three times weekly for 5 weeks, reduced VAS pain by 4.5 versus 3.0 units with sham and significantly increased grip strength, indicating clinically meaningful gains; a smaller pilot with 8 W over nine sessions alongside splinting and exercise showed no added benefit, possibly reflecting dose or co-intervention effects (Dundar Ahi and Sirzai, 2023). Carpal tunnel syndrome studies reported robust improvements in pain and nerve conduction, with one protocol reducing VAS from 6.8 ± 1.6 to 2.17 ± 1.48 (≈ 68% decrease) and shortening CMAP latency from 5.1 ± 0.51 to 4.2 ± 0.29 ms. In pregnant women with CTS, HILT improved pain (mean difference -2.48), grip strength (+4.11 kg), and sensory distal latency (-0.27 ms) relative to physiotherapy alone (Ezzati et al., 2020a). In SLE hand arthropathy, HILT plus routine therapy reduced VAS from 84.4 ± 7.12 to 35.6 ± 13.87 mm versus 88.4 ± 8.51 to 58.8 ± 10.54 mm in controls (between-group difference 23.2 mm; ES 1.9) and increased handgrip strength to 28.34 ± 8.3 kg versus 22.96 ± 8.76 kg, supporting favorable modulation of inflammatory and biomechanical components (Abdel-Aal et al., 2020).

##### 3.2.2.6 Face—pain reduction and mouth-opening improvements for temporomandibular joint disease

HILT targeting the face provided substantial pain relief and improved functional and quality*-*of*-*life in temporomandibular joint d*ise*ase (*TM*D), Bell’s palsy, and sinusitis. Pain was universally reduced in all *four* studies targeting *TM*D, while function and QoL were also improved in all cases where it was measured. In myogenic *TM*D, pain was decreased by 47*%* at *week* 4 in the HILT group *versus* 4% in sham controls (Ekici et al., 2021). For *TM*J *disc* displacement, HILT reduced pain by 48% at *week* 4, substantially outperforming TENS at 25% reduction *(Ekici et al., 2022*). In myofascial *TM*D pain, test groups receiving PBMT showed significantly lower pain scores compared *to* controls (p = 0.01) (*Nadershah et al., 2020*) A randomized trial using Piano*-*level Nd*-*YAG laser therapy for myogenic *TM*D reported significant pain reduction across all follow*-*up timepoints with no side effects (Qataya et al., 2025).

Most studies employed Nd:YAG lasers at 1,064 nm with high peak power (3 kW) and mean power of 10.5 W. The myogenic TMD study delivered 1,029.2 J per session over 15 sessions over 3 weeks (Ekici et al., 2021). For TMJ disc displacement, treatment involved 1,060 J total energy per 20-min session, performed 5 times weekly for 3 weeks (Ekici et al., 2022). The Bell’s palsy study using Nd:YAG HILO 3.0 delivered multi-wavelength therapy in a 4-session protocol just once per week (Abdullatif et al., 2022). A nonrandomized Bell’s palsy trial used dual wavelengths (808 and 905 nm) at 1.2 W power with 8.35 J/cm^2^ dosimetry over 72 sessions (3 times weekly for 6 months; Wu et al., 2023). The photobiomodulation TMD trial applied 940 nm laser at 7 W, delivering 300 J per treatment every 48 h for 10 days (Nadershah et al., 2020).

Beyond pain relief, studies documented functional improvements. Maximum mouth opening increased 27% in myogenic TMD versus 4% in controls at week 12, with significant gains in the JFLS-20 disability scale and OHIP-14 quality-of-life measures (Ekici et al., 2021). In TMJ disc displacement, HILT showed superior improvements in mouth opening (24% vs. 10% for TENS) and disability scores at both 4 and 12 weeks (Ekici et al., 2022; Qataya et al., 2025). For Bell’s palsy patients, significant improvements occurred in facial movement angles on both affected and non-affected sides (*p* < 0.05) (Abdullatif et al., 2022). Across all studies, no adverse effects were reported, establishing HILT as a safe, non-invasive modality. A sinusitis study found laser therapy combined with standard medical treatment superior to medical treatment alone across all variables (*p* < 0.001; Elkalla et al., 2020).

##### 3.2.2.7 Wounds—high-intensity laser therapy produces marked healing in cesarean and chronic wounds

HILT accelerates wound healing, relieves pain, and improves tissue quality when added to standard care, as shown in delayed cesarean section healing in diabetic women and in chronic refractory wounds. In diabetic women with delayed cesarean healing, pulsed 1,064 nm 10.5 W was applied for about 8 min per session in three phases, delivering a total of 360–540 J per treatment, three times per week for 6 weeks (Thabet et al., 2018). This regimen produced a 91% reduction in wound area (from 5.13 to 0.45 cm^2^) compared with ≈ 42% (4.98–2.90 cm^2^) under sham, a 41% versus 16% improvement in Pressure Sore Status Tool scores, and a significantly greater reduction in wound-related pain intensity, reflecting both faster tissue repair and clinically meaningful analgesia with HILT. In patients with chronic refractory wounds, adjunctive HILT was delivered using a 1,064 nm at 8 W in continuous biostimulation mode for 80 J/cm^2^, once daily, three times per week for 3 weeks, resulted in larger reductions in Bates-Jensen Wound Assessment Tool scores (-10.7 vs. -7.1; between-group difference 3.6 points) and PUSH scores (between-group difference 5.3 points), along with greater categorical wound-size reductions and no reported laser-related adverse events (Lu et al., 2021). Collectively, these parameter-defined protocols across distinct wound types support HILT as a clinically practical and effective adjunct that improves both the speed and comfort of wound healing beyond standard care alone.

### 3.3 Function and disability—high success rate for functional ability improvements (84%)

Across controlled clinical trials, HILT was consistently associated with superior functional outcomes versus control conditions, especially as an adjunct to structured rehabilitation. Function was measured with validated disability indices (WOMAC, SPADI, FIM, Oswestry, Neck Disability Index, Q-DASH, PRTEE, LEFS, SF-36 physical components) and performance-based tests (Timed Up and Go, stair-climb, 6-min walk, chair-stand), with most trials showing statistically and often clinically meaningful between-group differences favoring HILT.

Improvements in functional outcomes were observed in 48 of 57 studies (84%). Among 37 RCTs where HILT was the sole between-group treatment variable, 28 (76%) reported improved function, including better WOMAC scores and greater 6-min walk distances in conditions such as hemophilic arthropathy (El-Shamy et al., 2018; Akaltun et al., 2020).

In upper-extremity conditions, post-stroke shoulder trials found that HILT plus therapeutic exercise produced larger gains in functional independence and limb use than exercise alone, with greater improvements in FIM, shoulder ROM, SPADI, Brunnstrom stage, and quality-of-life scores (Korkmaz et al., 2022; Dajpratham et al., 2024). Trials in De Quervain’s tenosynovitis, lupus hand arthropathy, and lateral epicondylitis similarly showed superior functional recovery with HILT, reflected by larger increases in grip strength and greater reductions in upper-limb disability indices (Q-DASH, PRTEE, SF-36 physical function) versus sham laser, low-level laser, or extracorporeal shock-wave therapy (Karaca et al., 2022; Chongkriengkrai et al., 2023; Dundar Ahi and Sirzai, 2023; Bilir et al., 2024; Sen et al., 2024). An exception occurred in De Quervain’s tenosynovitis, where both groups received optimized splinting and exercise, which appeared to mask any incremental benefit of adding HILT (Chongkriengkrai et al., 2023).

Lower-extremity evidence, particularly in knee osteoarthritis, showed the most robust HILT–control separation: HILT plus exercise produced larger gains in WOMAC pain, stiffness, and physical-function subscales than exercise alone, low-level laser, ultrasound-based modalities, or ibuprofen phonophoresis, with HILT arms often achieving 60–65% total WOMAC reduction versus 30–40% in comparators. HILT groups also had greater improvements in knee flexion ROM, walking capacity (6-min walk), and performance tests (Timed Up and Go, stair-climb, 30-s chair-stand), and outperformed low-intensity pulsed ultrasound by nearly doubling percentage improvements in pain, ROM, proprioception, and WOMAC, although in at least one mild-to-moderate OA trial, intensive exercise plus sham produced similar functional gains to HILT (Laotammateep et al., 2025).

Trials in peripheral neuropathy and spine-related disorders also supported mobility and disability benefits: in diabetic neuropathy, HILT significantly shortened Timed Up and Go times versus sham, and in cervical radiculopathy, adding HILT to exercise or surgery yielded larger improvements in ROM and disability indices (Neck Disability Index, Oswestry Disability Index) (Chatterjee et al., 2019; Sherif et al., 2023; İnce et al., 2024). In systemic pain and complex regional conditions, HILT produced greater reductions in global disability than comparators, improving multidimensional health and function in fibromyalgia, Neck Disability Index and activity-based scores in chronic neck pain, Fugl-Meyer motor function in CRPS-I, SPADI in shoulder disorders (adhesive capsulitis, impingement), and generally LEFS and ROM in patellofemoral pain; meta-analytic patterns indicate that HILT’s functional benefits are most pronounced in conditions with substantial baseline impairment, such as post-stroke shoulder, moderate–severe knee OA, cervical/lumbar dysfunction, and lateral epicondylitis.

### 3.4 Range of motion—universal improvement in range of motion (100%)

Across 26 RCTs that explicitly measured range of motion (ROM), 25 showed improvement (≈96%). ROM increased in all 10 knee trials, 8/8 spine/back trials, 6/7 shoulder/arm trials, all 7 craniofacial/TMJ trials, and the single hand/wrist study.

In 10 knee osteoarthritis RCTs, photobiomodulation produced consistent, clinically relevant flexion gains, typically 5–15° over baseline; where quantifiable, HILT plus exercise outperformed sham or low-level laser, with Ahmad et al. reporting a 9.5° versus 3.1° flexion gain and a moderate between-group effect size (Ahmad et al., 2023).

Across eight spinal RCTs, adding HILT to rehabilitation significantly enhanced straight-leg raise and cervical flexion, extension, rotation, and lateral flexion versus physiotherapy, ultrasound, TENS, or exercise alone. In post-dissection cervical myofascial pain, HILT produced roughly 1.8–2.3-fold larger cervical ROM gains than exercise alone (Ahi and Sirzai, 2022).

Large absolute ROM gain was observed in the shoulder with rotator cuff tendinopathy, active abduction increased ≈39° (93°→132°) and passive abduction 31° (138°→169°), with parallel rotation improvements that persisted at 3–6 months while controls lost ROM (Elsodany et al., 2018). TMJ trials showed great relative gains (≈27% vs. 4% with placebo; ∼6.8-fold difference; Ekici et al., 2021). Across joints, HILT recipients generally maintained or further improved ROM at follow-up, whereas controls plateaued or declined, supporting a specific and durable effect of high-power photobiomodulation on ROM restoration.

### 3.5 Quality of life improvements with high-intensity laser therapy: comprehensive analysis across clinical conditions

HILT consistently improved health-related quality of life across diverse conditions, including cervical radiculopathy, diabetic peripheral neuropathy, fibromyalgia, knee osteoarthritis, osteoporotic hemiparesis, plantar fasciitis, post-stroke hemiplegic shoulder pain, and temporomandibular joint (TMJ) disorders.

In a randomized, double-masked, sham-controlled trial in older adults with painful diabetic peripheral neuropathy, quality of life improved significantly only in the HILT group (*p* = 0.001), not in sham controls, alongside larger pain reductions (Chatterjee et al., 2019). In myogenic TMJ disorder and TMJ disc displacement, HILT produced marked, sustained improvements in OHIP-14 scores at 4 and 12 weeks versus placebo HILT or TENS, indicating superior gains in oral-health–related quality of life and daily functions such as eating and speaking (Ekici et al., 2021, 2022).

For knee osteoarthritis, HILT outperformed low-level laser therapy on KOOS quality-of-life domains, with highly significant between-group differences (*p* < 0.001), reflecting greater improvements in pain, daily activities, sports participation, and knee-related QoL (Natalia et al., 2024). In women with fibromyalgia, HILT produced statistically significant benefits across all SF-36 domains (*p* < 0.001), demonstrating broad positive effects on physical functioning, mental health, vitality, and social participation (Kelini et al., 2025).

Post-stroke patients with hemiplegic shoulder pain showed significant Nottingham Health Profile gains with HILT, with greater QoL improvement than controls, supporting its role in enhancing recovery during critical rehabilitation periods (Korkmaz et al., 2022). In plantar fasciitis, both HILT and low-level laser improved pain and FAOS QoL scores, but HILT had more pronounced effects; SF-36 improvements at 3 months were significantly greater with HILT than with ESWT alone, suggesting superior long-term QoL benefits (Ordahan et al., 2018).

Among osteoporotic hemiparetic patients, QUALEFFO-41 scores improved significantly in both HILT and ESWT groups versus control, with HILT providing the largest QoL gains, a key advantage in a highly vulnerable, fracture-prone population (Abo Elyazed et al., 2023). In cervical radiculopathy, SF-36 subscales improved in all groups, but high-intensity laser plus exercise produced the greatest medium-term QoL benefits compared with placebo laser plus exercise or exercise alone, underscoring the additive value of HILT as a rehabilitation adjunct (İnce et al., 2024).

Taken together, these trials show that HILT not only reduces pain but also produces statistically robust and clinically meaningful improvements in multidimensional quality-of-life measures, making it a high-value option for enhancing overall wellbeing and functional capacity in chronic musculoskeletal and neurologic conditions.

### 3.6 HILT exploratory—effects beyond pain relief, ROM, function/disability, and/or QoL

HILT was additionally evaluated across a surprisingly broad set of non-pain outcomes, with controlled trials supporting that appropriately dosed, high-power protocols can induce measurable structural and functional changes in multiple systems, including body composition and lung function in obesity, myocardial injury markers in acute coronary syndrome, working memory and frontal connectivity in mild cognitive impairment, nerve conduction in entrapment neuropathies, near-elimination of pelvic pain together with reduction in graded endometrial adhesions, bone mineral density in osteopenia/osteoporosis, cartilage and muscle thickness in degenerative joint disease and tendon tears, and the rate and quality of tissue repair in both acute and chronic wounds.

#### 3.6.1 HILT produces major improvements in pain and adhesions in endometriosis

HILT yielded remarkably positive outcomes in women with endometriosis, with most participants achieving near-elimination of pain, marked reductions in laparoscopically graded adhesions, and clinically meaningful gains in health-related quality of life. Endometriosis is a common, chronically painful gynecologic disease affecting roughly one in ten women of reproductive age, and existing medical and surgical options often provide only temporary relief and substantial side effects, leaving many women with persistent symptoms and profound distress related to threatened or lost fertility.

In this RCT, 40 women with mild or moderate laparoscopically confirmed endometriosis received either HILT plus standard medroxyprogesterone or sham laser with the same regimen for 8 weeks. The protocol delivered 1,300 J per session at 52.5 W/cm^2^, three times weekly, scanning over the suprapubic and iliac fossa regions with contact irradiation. HILT produced striking benefits: 90% of women started with severe or unbearable pain, yet after treatment 85% reported no or only mild pain, whereas in the control group 75% still reported at least moderate pain and 45% continued to have severe pain. Likewise, 85% of HILT-treated women reported complete or excellent pain relief compared with only 20% of controls, with parallel shifts in the HILT arm from predominantly moderate (60%) to exclusively minimal or mild endometriosis (100%) and broad EHP-5 quality-of-life improvements. This study indicates that properly dosed HILT is a highly promising nonpharmacologic adjunct for reducing pain and adhesions and restoring daily function in women with endometriosis, meriting broader clinical use alongside larger, multicenter trials to confirm durability and refine dose parameters.

#### 3.6.2 Brain—high-power transcranial HILT shows promise for cognitive enhancement

Only one controlled trial focused on the brain, evaluating transcranial HILT as a treatment for mild cognitive impairment, and it used a high-power 1,064 nm class 4 laser delivering 3.4 W to the left dorsolateral prefrontal cortex at a power density of 0.25 W/cm^2^ for 18 min, yielding a total fluence of ∼3,700 J to the target (Han et al., 2025). This protocol produced statistically significant and clinically meaningful gains in working memory in the tPBM-only arm, with the largest effect seen when high-frequency TMS was added on top of HILT and was accompanied by increased functional connectivity between the lateral premotor cortex and right DLPFC. Compared with the broader mild cognitive impairment PBM literature, dominated by lower-power near-infrared LEDs or class 3B lasers with mixed effect sizes and more modest network-level changes, this single high-power 1,064 nm study stands out for both the magnitude of its working-memory improvement and the clear connectivity signature in fronto-premotor networks, suggesting that higher irradiance, deeper-penetrating wavelengths, and targeted DLPFC dosing may yield stronger cognitive effects than typical low-level tPBM protocols.

#### 3.6.3 Bones—sustained improvements in bone mineral density

One study examined the effects of HILT on bone mineral density in men with osteopenia or osteoporosis. The scan was performed longitudinally and transversely in the lower back area to cover the lumbar vertebrae, paraspinal muscles, and upper part of the gluteus maximus. The laser was also applied to the anterolateral, lateral, and posterolateral aspects of the proximal hip region. The study demonstrated a medium effect size (ES = 0.5), and most importantly, this effect size was maintained even 1 year after the treatment was stopped (ES = 0.4; Alayat M. S. M. et al., 2018). The treatment used in this study involved the administration of micropulsed light with an average power of 10.5 W (at 50 W/cm^2^, 1,064 nm, 120–150 μs, 10–30 Hz, 3,000 J per session using HIRO 3.0) three times per week for a total of 24 weeks.

#### 3.6.4 Abdomen—large effect on pulmonary function with moderate weight reduction

One study evaluated high-intensity laser therapy combined with aerobic exercise for treating obesity and associated pulmonary dysfunction. The abdominal treatment area was demarcated in supine position as a quadrilateral bordered superiorly by the 10th costal cartilages and inferiorly by the anterior superior iliac spines, with vertical lines connecting these landmarks bilaterally. In patients with BMI > 30 and waist circumference > 102 cm, HILT (52.5 W/cm^2^, 1,064-nm, 10.5 W, 0.1% duty cycle) was applied twice weekly for 12 weeks (Abdelaal, 2023). Compared with placebo, the HILT group showed significantly greater improvements in body weight, BMI, waist circumference, forced vital capacity (FVC), and FEV*1*. HILT produced a large effect size for FVC (ES = 1.3) and medium effect sizes for reductions in body weight (ES = 0.3) and waist circumference (ES = 0.6).

#### 3.6.5 Heart—HILT reduces reperfusion injury in acute coronary syndrome

One study focused on the heart. Treatment targeted three pericardial sites: left parasternal intercostal spaces 2–3 and apex. In patients with acute coronary syndrome undergoing primary percutaneous coronary intervention (pPCI), Class IV laser therapy significantly reduced troponin I elevation (a marker of myocardial injury) compared to sham control (*p* = 0.02), with no adverse effects reported. Left ventricular ejection fraction (LVEF) showed no significant difference between groups. The reduced troponin I elevation indicates that laser therapy limits reperfusion injury, the paradoxical damage that occurs when blood flow is restored to ischemic heart tissue during pPCI. By attenuating this secondary injury, adjunctive laser therapy may reduce the total extent of myocardial damage during acute coronary events, potentially preserving more viable heart muscle. This is particularly relevant in coronary syndrome management because reperfusion injury can account for up to 50% of final infarct size despite successful vessel reopening. The feasibility and safety profile demonstrated here suggests laser photobiomodulation could be integrated into standard interventional cardiology protocols to improve outcomes in patients undergoing emergency coronary procedures, though the lack of difference in LVEF suggests that larger trials or longer follow-up may be needed to detect functional cardiac benefits.

### 3.7 Connective tissue high-intensity laser therapy promotes cartilage thickening

Across musculoskeletal conditions, randomized trials suggest that appropriately dosed, higher-power HILT can induce modest but measurable structural remodeling of cartilage, fascia, and tendon on imaging, most convincingly in Akaltun et al.’s femoral cartilage trial and Kumar et al.’s lumbar canal study, while lower-dose or less frequent protocols more often yield equivocal or exercise-attributable tissue changes and predominantly symptomatic benefits.

### 3.8 Sustained femoral cartilage gains following HILT

Three clinical studies evaluated femoral cartilage thickness in knee osteoarthritis patients before and after treatment with HILT. Among these, the trial by Akaltun et al. stands out for demonstrating robust and regionally consistent structural change (Akaltun et al., 2020). Using ultrasonography, the authors measured femoral cartilage at the medial condyle, intercondylar area, and lateral condyle femoral cartilage and consistently observed clinically significant increases in thickness at all three sites at 6-week follow-up in the HILT + exercise group (*p* < 0.05). The magnitude of improvement was substantial, with Cohen’s *d* indicating a large effect for medial femoral cartilage (ES = 1.09) and medium effects for the intercondylar (0.66) and lateral (0.76) femoral cartilage regions. Importantly, these gains were greater at 6 weeks than immediately post-treatment, consistent with sustained tissue regeneration rather than transient edema.

These divergent outcomes likely reflect differences in laser dosing and scheduling. Akaltun et al. used a 1,064 nm Nd:YAG device at higher power (up to 12 W) in a dual-phase protocol delivering 300 J analgesic followed by 3,000 J biostimulation over 10 sessions performed 5 days per week for 2 weeks, whereas Ezzati et al. applied a much lower power (1.6 W, 5-min sessions) and Ekici and Ordahan used a mixed 10W/5W regimen over only nine sessions performed every other day. Taken together, the available data cautiously supports that higher-power, higher-dose, and more regularly scheduled daily HILT is more likely to provide measurable and sustained cartilage-thickness gains than the lower-intensity, lower dose, less regular treatment plans.

### 3.9 Significant within-group enlargement of lumbar canal diameter following HILT

A study investigating HILT in lumbar disc herniation (LDH) demonstrated significant morphological changes in the lumbar spinal canal (Kumar et al., 2025). One primary outcome was the anteroposterior (AP) diameter of the L5-S1 canal, assessed by MRI. In the experimental group, which received HILT at 9 W (3,780 J over 7 min, three times weekly for 10 weeks), the mean AP canal diameter increased from 12.77 ± 2.99 mm to 12.93 ± 3.00 mm, representing a statistically significant within-group improvement (Wilcoxon z = 4.154, *p* < 0.001). By contrast, the sham-treated control group showed essentially no change in canal diameter (12.10 ± 1.64 mm to 12.09 ± 1.63 mm; z = 0.16, *p* = 0.873), indicating that spontaneous recovery or placebo effects were unlikely to account for the observed structural changes. However, between-group comparison of post-treatment values using the Mann-Whitney U test did not show a statistically significant difference, suggesting that although HILT produced a significant increase in spinal canal diameter relative to baseline, the absolute magnitude of change was modest. These findings, together with known photobiomodulatory effects of Class IV laser on inflammation, tissue repair, and matrix remodeling, support the potential of HILT to induce measurable structural improvement in the lumbar canal in LDH, while highlighting the need for larger, longer-term trials to determine the durability and clinical significance of these morphological effects.

By contrast, the other two studies reported more limited or less attributable structural effects. Ezzati et al. found that HILT produced a greater short-term increase in vastus medialis muscle thickness than low-level laser therapy after 10 sessions over 2 weeks, but group differences had disappeared by 1-month follow-up (Ezzati et al., 2024). Ekici and Ordahan observed statistically significant increases in medial and intercondylar femoral cartilage thickness in both the HILT + exercise and sham laser + exercise groups after treatment and at 3 months, with no significant between-group differences at any time point, suggesting that exercise, rather than HILT *per se*, drove the cartilage changes in that trial (Ekici and Ordahan, 2023).

### 3.10 Plantar fasciitis and deep tissue structure

Plantar fascia thickness changes were assessed across three randomized controlled trials (Jitpimolmard et al., 2026). A plantar fascia thickness of > 4 mm by ultrasonographic imaging supports the diagnosis (Draghi et al., 2017). The Jitpimolmard trial using 1,064 nm wavelength at 12 W documented plantar fascia thickness reduction (mean difference –0.02 mm), while the Naruseviciute study reported thickness reduction of 0.30 mm over 3 weeks (Naruseviciute and Kubilius, 2020). The Thammajaree investigation measured plantar fascia and flexor digitorum brevis thickness using reliable ultrasound imaging (ICC 0.920), documenting structural changes with modest magnitude (Thammajaree et al., 2023). Across these studies, plantar fascia thickness reductions were observed, potentially reflecting changes in inflammatory tissue status or tissue organization, though the clinical significance of these magnitude changes remains to be established.

### 3.11 Shoulder tendon remodeling—divergent outcomes

Shoulder tendon structural changes varied between treatment approaches (Zaki et al., 2022). The Zaki investigation demonstrated that HILT combined with kinesiology taping produced significant therapeutically favorable supraspinatus tendon thickness reduction in both short and long axes, accompanied by increased echogenicity. Using 4W over seven sessions, this protocol was associated with supraspinatus tendon thickness changes and increased echogenicity compared to low-level laser or sham treatment groups.

In contrast, the Uzun study of partial supraspinatus tears documented no significant changes in supraspinatus tendon thickness, despite measurable reductions in pain and functional disability scores (Uzun et al., 2025). The divergence in structural outcomes between studies may reflect differences in baseline pathophysiology. Zaki’s subacromial impingement syndrome cohort presented with inflammatory tendinopathy without full-thickness structural defects, whereas Uzun’s cohort had partial-thickness tears representing structural discontinuity. The absence of tendon thickness changes in the partial tear population despite clinical outcome improvements suggests that pain and functional gains may occur through mechanisms independent of structural tissue remodeling. Clinical improvements were documented in both pathologic subtypes, indicating that therapeutic effects may operate through multiple physiologic pathways.

### 3.12 Safety profile

No serious adverse events (AEs) were observed in any of the 105 randomized controlled trials (RCTs) comprising 2,710 participants exposed to class IV high-intensity laser photobiomodulation therapy, including 0.5 W protocols. When individuals with rare genetic hemostatic disorders are excluded, the safety signal becomes more striking: among participants without known genetic disease (e.g., those treated for lumbar disc herniation, adhesive capsulitis, or de Quervain’s tenosynovitis), only about 1 out of 277 exposed individuals experienced any AE, corresponding to approximately 0.36% (10 of 2,710 patients treated with HILT), meaning 99.6% had no detectable adverse reaction.

When all participants are considered, including those with genetic hemostatic disorders, the overall AE rate remains low: across all 105 RCTs, only 15 participants (0.55%) experienced treatment-related AEs, and every event was mild, transient, and self-limiting. In the adhesive capsulitis trial, one light-skinned female developed a photoallergic reaction to HILT that resolved after discontinuation and did not produce systemic sequelae (Atan and Bahar-Ozdemir, 2021). In de Quervain’s tenosynovitis, one HILT-treated participant had mild wrist swelling that resolved with local cold therapy, and two sham-treated participants reported transient post-treatment pain that subsided spontaneously; the authors explicitly state that no serious AEs occurred (Chongkriengkrai et al., 2023). In the lumbar disc herniation RCT, AEs in the HILT arm were limited to transient erythema at laser sites, mild paraspinal muscle spasms, and brief headaches, all resolving within 72 h without medication, with no burns, neurologic deficits, or other serious complications (Song et al., 2025).

The most common AE was transient erythema at the laser application site. Clinically, transient erythema is a short-lived, localized area of skin redness due to vasodilation and increased cutaneous blood flow induced by the laser’s photothermal and photobiomodulatory effects. It appears as a discrete red patch without blistering, ulceration, or significant warmth and, in the reported trial, resolved within 72 h using only local cooling measures, without analgesics or treatment interruption. Other reported AEs, mild edema at the treatment site, short-lived muscle spasm, and transient post-treatment pain, also resolved spontaneously or with simple non-pharmacologic measures and did not require withdrawal or medical treatment beyond observation.

Interestingly, individuals with hemophilia A and hemophilic arthropathy contributed disproportionately to the small pool of observed AEs: integrating the pediatric RCT in hemophilic arthropathy with prior pilot work, three of five AEs in the four trials occurred in patients with hereditary bleeding disorders, yet even in this vulnerable population, HILT was described as safe, and no bleeding events, worsening arthropathy, or factor-related complications were attributed to laser exposure (El-Shamy and Abdelaal, 2018). Taken together, these 105 RCTs, spanning musculoskeletal, post-procedural, and hemophilic populations, indicate that class IV HILT at or around 0.5 W (and including higher-power 8–15 W protocols) is associated with a low incidence of minor AEs, no serious AEs, and no signal for organ toxicity, neurologic injury, or treatment-related mortality. When contrasted with standard pharmacotherapeutic approaches for chronic pain and musculoskeletal conditions, where constipation, sedation, dependence, and withdrawal are common with opioids, and serious events such as gastrointestinal bleeding, hepatotoxicity, renal injury, and even death are well-documented with NSAIDs, acetaminophen, and centrally acting agents, the emerging safety profile of HILT suggests a risk level orders of magnitude lower, with the “typical” adverse experience being a self-limited patch of redness rather than systemic toxicity, dependence, or life-threatening organ failure.

### 3.13 HILT vs. LLLT

HILT was found to be superior to LLLT in all 13 studies, with a statistically significant effect difference observed in 12 of them (Table 4). HILT demonstrated superiority over LLLT in the treatment of knee osteoarthritis (*k* = 5), plantar fasciitis (*k* = 2), subacromial impingement (*k* = 2), carpal tunnel syndrome (*k* = 2), lateral epicondylitis, and adhesive capsulitis (frozen shoulder). From 9 studies limited to end-of-treatment pain measures, a total of 254 subjects in the HILT cohort and 256 subjects in the control cohort yielded a SMD of -1.35 with a 95% confidence interval of -1.88 to -0.83 and a significance, *p* < 0.05 (Figure 6). The *I*^2^ value indicated that 85% of the variability among studies arises from heterogeneity rather than random chance.

**TABLE 4:** Summary of HILT versus LLLT studies included in this meta-analysis.

References	HILT			LLLT			Superiority			Cohen’s *d* effect size
	Power or power density	λ (nm)	Dosage (J)	Power or power density	λ (nm)	Dosage (J)	Pain reduction	Function	QoL	
Knee osteoarthritis (Ahmad et al., 2022)	5 W	1,064	3,190	0.4 W	830	400	HILT	HILT	HILT	1.3
Carpal tunnel syndrome (Ezzati et al., 2019)	1.6 W	808	?	50 mW	860	?	HILT	HILT nerve conduction action potential		1.6
Knee osteoarthritis (Ezzati et al., 2024)	1.6 W	808	?	50 mW	808	?	HILT	HILT		
Carpal tunnel syndrome (Hojjati et al., 2020)	5 W	1,064	?	500 mW	910	?	HILT	HILT		
Lateral epicondylitis (Kaydok et al., 2020)	8 W/cm^2^	1,064	?	240 mW	904	?	HILT	HILT SF-36 and QDASH	HILT	0.4
Knee osteoarthritis (Koevska et al., 2025)	4 W	?	?	200 mW	?	?		HILT		1.3
Plantar fasciitis (Naruseviciute and Kubilius, 2020)	7 W 7 W/cm^2^	1,064	3,000	50 mW	785	140	Both groups exhibited reduced pain. there was no untreated control.			0.2
Knee osteoarthritis (Natalia et al., 2024)	10 W and 5 W	1,064	?	500 mW	910	?	HILT	HILT		1.7
Adhesive capsulitis (Ordahan et al., 2023)	12 W/cm^2^	1,064	?	240 mW	904	?	HILT			2.2
Plantar fasciitis (Ordahan et al., 2018)	6 W/cm^2^	1,064	?	240 mW	904	?	HILT	HILT heel tenderness	HILT foot and ankle outcome scores	2.2
Subacromial impingement syndromes (Saleh et al., 2025)	4 W	810 and 980	300	200 mW	850	60	HILT	HILT	HILT	5.3
Knee osteoarthritis (Sen et al., 2024)	?	1,064	3,00 4 tx 3,000 6 tx	800 mW	1,064	1,250	HILT	HILT		
Knee osteoarthritis (Wibisono et al., 2024)	10 W 10 W/cm^2^	1,064	12 and 120 J/cm^2^	78 mW	905	1.5 J/spot 6 spots	HILT superior in restoring balance in knee osteoarthritis			
Subacromial impingement syndrome (Zaki et al., 2022)	4 W 5 W/cm^2^	810 + 980	2,050	200 mW 250 W/cm^2^	810 + 980	2,000	HILT	HILT		

The table presents laser parameters and relative clinical outcomes. Across studies, HILT generally demonstrates greater improvements in pain and function than LLLT, suggesting a potential benefit of higher-intensity dosing for laser therapy. Cohen’s *d* values are restricted to end-of-treatment comparisons between HILT and LLLT to allow the most comparable effect size estimates across studies. Effect sizes are reported only for studies that provided the requisite data (SMDs and SDs) needed to calculate ESs.

### 3.14 Clinical benefit in knee osteoarthritis depends on high-intensity, not low-power, photobiomodulation

Across the five KOA trials, comparison of HILT versus LLLT directly addressed whether sufficient power is required for photobiomodulation to reach and modulate deeper joint tissues. HILT protocols uniformly used powers in the 4–12 W range at predominantly 1064 nm, delivering energy in the *kilojoule* range per session, whereas LLLT protocols relied on sub-watt outputs (typically 0.2–0.8 W) and per-session doses of only a few hundred to about 1,250 J (Table 4). In every KOA study, these higher-power, higher-energy HILT conditions were associated with superior clinical outcomes, supporting the view that adequate power is a prerequisite for effective deep-tissue photobiomodulation in knee osteoarthritis.

Ahmad used 5W at 1,064 nm and delivered 3190J per session, while the comparator LLLT arm used 0.4 W at 830 nm and 400 J per session (Ahmad et al., 2023). This roughly 10-fold energy difference, coupled with more than an order-of-magnitude higher power, translated into clear superiority of HILT for pain, function, and mobility, with a large effect size (ES = 1.3) favoring HILT on end-of-treatment VAS pain. Sen’s KOA trial employed an Nd:YAG-class HILT regimen that ramped from 300 J in early sessions to 3,000 J in later sessions (up to 12 W, 1,064 nm), whereas the LLLT arm used 800 mW with 1,250 J per session (Şen et al., 2025). Despite LLLT delivering a moderate dose by conventional standards, the much higher power and total energy of HILT were again accompanied by superior pain and functional outcomes, reinforcing a dose-response relationship between deep-tissue energy deposition and clinical benefit.

Koevska delivered KOA HILT at 4 W versus 200 mW in the LLLT arm, a ∼20-fold power difference with comparable treatment times (Koevska et al., 2025). Even though total joules were incompletely reported, the higher average power indicates substantially greater energy delivery into the joint, which is reflected in a large effect size (ES = 1.3) for function outcomes at end of treatment. Natalia’s protocol combined 10 W and 5 W phases at 1,064 nm for KOA, contrasted with 500 mW at 910 nm in the LLLT arm, and again favored HILT with a very large effect (ES = 1.7) on VAS pain (Natalia et al., 2024). Wibisono focused on balance and dosed HILT at 10 W with fluences up to 120 J/cm^2^ over 25 cm^2^, while LLLT used only 78 mW and 1.5 J per spot; this high-power, broad-field irradiation produced superior recovery of balance in KOA, consistent with the need to energize deeper neuromuscular structures, not just superficial tissues (Wibisono et al., 2024).

Taken together, the KOA subset in Table 4 shows a consistent pattern: high-intensity protocols using 4–12 W and kilojoule-level doses at 1,064 nm not only deliver substantially more energy to deeper tissues than sub-watt LLLT, but also yield moderate-to-very-large superiority effect sizes for end-of-treatment VAS pain (Cohen’s *d* in the large effect size range of roughly 1.3–1.7 where calculable). This convergence of parameter contrast (power and joules) with magnitude of effect strongly supports that, in knee osteoarthritis, high-intensity dosing is not simply a technical detail but a key determinant of whether photobiomodulation meaningfully reaches and benefits deep articular and periarticular tissues.

### 3.15 High-intensity laser provides greater symptom relief than low-level laser in SAIS

Across two randomized trials in SAIS, HILT consistently outperformed LLLT (Zaki et al., 2022; Saleh et al., 2025). In Saleh et al., 42 patients received exercises plus either HILT (810/980 nm) or LLLT (904 nm), three times weekly for 3 weeks; both lasers improved pain and function, but HILT produced much larger gains in SPADI pain, disability, total score, PPT, and sleep quality, with large effect sizes favoring HILT. Zaki et al. treated 30 SAIS patients with kinesiology taping plus either high-power laser, low-level laser, or sham; all groups improved, yet only the high-power laser group showed significant advantages in pain reduction, SPADI subscales, supraspinatus tendon thickness, occupation ratio, and echogenicity.

### 3.16 High-power laser shows favorable trends over low-level laser in CTS management

Across two randomized CTS trials, laser therapy improved pain and nerve function, with the strongest motor improvements seen with carefully dosed HILT (Hojjati et al., 2020; Ezzati et al., 2024). Ezzati et al. (2024) randomized 98 patients to exercise alone, LLLT, or HILT variants over five sessions; all groups improved, but 1.6 W HILT produced the greatest reduction in VAS pain and the largest gains in median CMAP latency and amplitude. Hojjati et al. treated 45 patients with splinting alone, low-power laser, or high-power laser and found all three arms improved, with both laser groups outperforming splinting and HPL showing numerically greater, though not statistically superior, improvements versus LPL.

### 3.17 Plantar fasciitis: favorable outcomes with high-intensity laser therapy

Across two randomized trials in plantar fasciitis, both HILT and LLLT produced meaningful pain relief and functional gains, but the relative advantage of HILT depended on dosing strategy (Ordahan et al., 2018; Naruseviciute and Kubilius, 2020). Ordahan et al. used a 12 W, 1,064 nm Nd:YAG HILT protocol (initial 8 W, 6 J/cm^2^ analgesic phase followed by 6 W, 120–150 J/cm^2^ biostimulatory sessions; nine treatments over 3 weeks) versus 904 nm LLLT delivering 8.4 J at the insertion plus 8.4 J along the fascia, three times weekly. HILT yielded larger improvements in VAS pain, heel tenderness, and FAOS pain and function than LLLT. In contrast, Naruseviciute and Kubilius compared eight sessions of HILT versus LLLT over 3 weeks and found similar reductions in pain, pressure-pain thresholds, and plantar fascia thickness, with only patient-rated efficacy favoring HILT (73% vs. 51% reporting > 50% improvement), underscoring that higher-power, longer-dose HILT regimens may be needed to reveal clear superiority.

### 3.18 High-intensity laser yields superior shoulder and elbow outcomes

Across two RCTs in upper-limb disorders, both HILT and LLLT improved pain and function, with several outcomes favoring high-power protocols (Ordahan et al., 2018; Kaydok et al., 2020). Kaydok et al. (2020) treated lateral epicondylitis using 1,064 nm HILT (three 75 s sessions at 8 W, 6 J/cm^2^, 150 J total, followed by six 30 s sessions at 6 W, 120–150 J/cm^2^; nine sessions over 3 weeks) versus 904 nm LLLT at 240 mW (2.4 J/cm^2^ to six points, 30 s each; three sessions weekly). Both groups improved, but HILT produced greater gains in hand-grip strength, QDASH, and SF-36 physical scores. Ordahan et al. used a similar nine-session HILT protocol (8 W, 10 J/cm^2^ then 12 W, 120 J/cm^2^) versus 27 J/session LLLT in adhesive capsulitis; combined with stretching, both reduced VAS and SPADI, with significantly larger improvements under HILT.

### 3.19 Risk of bias assessment

The risk of bias are presented in Figure 7 and in a traffic plot Supplementary Figure 2. A majority of studies demonstrated a high risk of performance bias. Randomized initial selection was applied in 103 of 105 studies. The principal source of performance bias was the lack of blinding among researchers administering the interventions (78%). Blinding status could not be determined in 6 studies. Roughly half (51%) of studies were assessed as having a low risk of detection bias; however, approximately one-quarter (24%) exhibited a serious risk, with blinding status similarly indeterminate in six studies.

**FIGURE 7:**
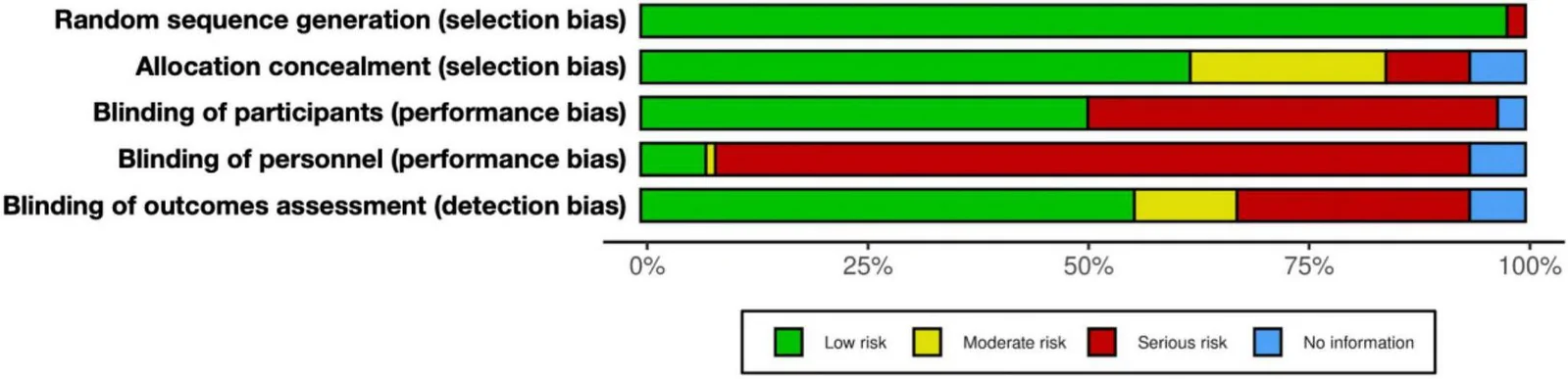
Risk of bias summary graph representative of authors’ judgements for each study.

Two triple-blinded studies exhibited a low risk of bias, both reporting large effect sizes. In knee osteoarthritis, high-intensity laser therapy produced a large effect at end-of-treatment (ES = 4.0) and maintained a substantial effect at 12-week follow-up (ES = 3.0) following administration of 4 W/cm^2^ of 1,,064 nm light three times weekly for 4 weeks (Nazari et al., 2019). For thumb arthritis, HILT resulted in a large end-of-treatment effect size (ES = 1.2), which decreased to a medium effect (ES = 0.5) at 3 months post-treatment (Cantero-Téllez et al., 2019).

### 3.20 Certainty of evidence

The certainty of evidence for outcomes was evaluated using the GRADE framework. The assessment results are summarized in Table 5.

**TABLE 5:** GRADE assessment: summary of findings and certainty of evidence.

Outcome	Summary of findings	Certainty of evidence (GRADE)
Pain intensity	HILT reduced pain across major anatomical regions, with large end-of-treatment effects and persistent benefit at follow-up.	Moderate
Range of motion	ROM improved in 25 of 26 trials, with consistent gains across knee, TMJ, spine/back, and shoulder outcomes.	Low to moderate
Serious adverse events	No serious adverse events were reported; only rare, transient local reactions were described.	High

This GRADE assessment is based on evidence synthesized from the primary studies included in the meta-analysis.

For pain intensity (global and by anatomical region), the certainty of evidence was rated as moderate. HILT produced large and durable reductions in pain with standardized mean differences around 1.0 for global pain and 1.2–1.6 for knee, shoulder/arm, and spine categories, and confidence intervals generally excluded no effect. However, many contributing trials were small, single-center studies with variable blinding and heterogeneous co-interventions, leading to downgrading for risk of bias and inconsistency despite low indirectness and only moderate concern about publication bias.

Range of motion (ROM) was evaluated in 26 randomized trials, with improvement observed in 25 (96%), including all knee and temporomandibular joint trials and most spine and shoulder trials. Because ROM is an objective, clinician-measured outcome and the direction of effect was highly consistent, certainty was judged as low-to-moderate: the body of evidence was downgraded for study limitations and imprecision but positioned higher than composite functional outcomes, which showed more variable magnitude of benefit.

Safety outcomes achieved the highest GRADE rating. Across 105 controlled trials involving 2,710 HILT participants and 2,925 controls, no serious adverse events were reported, and only rare, transient local reactions were described. Devices and dosimetry spanned clinically relevant class IV HILT parameters (average power about 9 W, up to 30 W, with power densities up to 52.5 W/cm^2^), making indirectness low and supporting high-certainty evidence that clinically important serious harm is very unlikely with the HILT protocols evaluated in these trials.

## 4 Discussion

The objective of this meta-analysis was to begin to evaluate the full range of indications that can benefit from high-powered laser photobiomodulation therapy. The secondary objective was to determine the optimal laser settings and treatment regimens based on the currently available data from randomized controlled trials (RCTs). Pain levels after high-powered laser therapy (HILT) were the most commonly measured outcome, and all studies focused on the knee, spine, shoulder/arm, and wound showed reductions in pain. Most of these reductions were significant, as indicated by the large effect sizes (Tables 1, 2). Additionally, improvements in range of motion, mobility, quality of life, cartilage growth, rotator cuff tear recovery, and nerve conduction were also demonstrated.

Consistent with our hypothesis, all 13 randomized trials directly comparing HILT with LLLT found superior outcomes with the higher-powered lasers. The only partial exception was a plantar fasciitis trial in which both high- and low-energy PBMT reduced pain, possibly because treatment was not targeted to the true pathogenic anatomy (see Limitations; Table 4). Four of these HILT-superior studies involved shoulder/arm disorders, with others focused on the knee, foot, and hand. In double-blind RCTs on myofascial pain and TMJD, LLLT failed to reduce pain, whereas matched HILT protocols produced significant analgesia (Shobha et al., 2017; Nadershah et al., 2020). Overall, our 6-year meta-analysis indicates that high-power laser PBMT consistently outperforms LLLT.

### 4.1 Consistency of HILT evidence across meta-analyses

Our findings are in agreement with *all* prior meta-analyses (Abdildin et al., 2023; Arroyo-Fernández et al., 2023; de la Barra Ortiz et al., 2023; Silva et al., 2023; Xie et al., 2023; Poenaru et al., 2024). Very high rates of positive outcomes (96%) were observed after a comprehensive evaluation of HILT treatment specifically targeting pain (July 2020 to August 2022; *k* = 31 RCTs; Silva et al., 2023). HILT combined with exercise therapy (HILT + ET) outperformed LLLT + ET in knee osteoarthritis up to December 2019 (*k* = 10 RCTs), and a subsequent 2024 meta-analysis of RCTs from 2009 to 2024 (*k* = 23) likewise concluded that HILT was significantly more effective than LLLT (Ahmad et al., 2022; Poenaru et al., 2024). An analysis focused on knee osteoarthritis concluded that HILT effectively alleviates pain (≤ September 2022; *k* = 9 CTs; Cai et al., 2023). Likewise, an analysis of HILT treatment for neck pain concluded that it effectively reduces pain and improves cervical ROM (≤ March 2022; *k* = 9 RCTs; Xie et al., 2023). Another analysis comparing HILT to other physical therapy modalities for knee osteoarthritis found that HILT is superior in terms of pain reduction and functional improvement (≤ March 2022; *k* = 10 RCTs). In line with our observations, an extensive meta-analysis of musculoskeletal outcomes (≤ September 2022; *k* = 42 RCTs) found large (> 0.8) effect sizes in pain reduction and improved function after HILT treatment (Arroyo-Fernández et al., 2023). Overall, it is evident that HILT holds significant potential for transforming the treatment of various musculoskeletal pains, stiffness, and loss of function. However, there are current limitations to implementation, including a general lack of physician awareness of the wide-ranging therapeutic utility of HILT and restricted patient access.

### 4.2 High-intensity laser therapy delivers durable, clinically significant benefits

The persistence of comparable effect sizes at both the end of treatment and extended follow-up indicates that high-intensity laser therapy can act on the underlying pathological processes, providing a more durable and clinically meaningful therapeutic effect rather than merely transient symptomatic relief (Figure 2). Between-study analysis of heterogeneity of outcome metrics at end of treatment versus later follow-up yielded *I*^2^ values of 86 and 89%, respectively, for knee studies (*k* = 21), 81 and 82% for spine studies (*k* = 13), no meaningful change in heterogeneity for shoulder/arm studies, and *I*^2^ values of 84 and 82% for trials comparing HILT versus LLLT (*k* = 10). These findings indicate that treatment effects are not attributable to chance alone and support the conclusion that benefits are durable and sustained rather than transient. In clinical terms, this is consistent with a modality that contributes to lasting tissue and/or nociceptive pathway changes that address the core drivers of musculoskeletal pain and dysfunction, rather than merely providing transient symptomatic relief.

### 4.3 High-powered laser PBMT as a low-toxicity alternative to NSAIDs and opioids for musculoskeletal pain

Out of the 2,710 patients treated with HILT in this meta-analysis, not a single serious adverse event was reported. This represents a markedly better safety profile than the pharmacotherapies commonly used for chronic pain. In addition, our GRADE assessment rated the certainty of evidence for serious adverse events as high, reflecting consistent reporting across 105 controlled trials and the absence of any clinically important harms with class IV HILT dosimetry. By contrast, opioid therapy often produces tolerance, addiction, constipation, nausea, and potentially fatal respiratory depression, while doing nothing to correct the underlying pathology (Paul et al., 2021).

In fact, opiate-related overdoses have been among the fastest rising causes of death in America, rising to epidemic proportions over the past three decades from 1988 until 2018 (CDC, 2021 CDC’s Response to the Opioid Overdose Epidemic | CDC’s Response to the Opioid Overdose Epidemic | CDC, 2021; National Institute on Drug Abuse, 2023) By 2028, the estimates are that more Americans will have died in the 21st century of drug overdoses than those who died in all of America’s wars over its entire history. Additionally, a 2022 report released by the U.S. Commission on Combating Synthetic Opioid Trafficking estimates that drug overdoses now cost the U.S. approximately $1 trillion every year (Forbes, 2024 Porterfield, n.d.). By comparison, no serious adverse events have ever been reported, let alone deaths, when using high-powered PBMT. High-powered laser PBMT should be considered as an approach that may enable the transition from chronic medication to a tolerable or potentially even painless health status.

Most significantly, high-powered laser PBMT RCTs provides sustained benefits in bio-stimulation of tissue healing (Figure 2) with regeneration, unlike pharmaceuticals commonly used to address musculoskeletal disorders, including the chronic use of corticosteroids and NSAIDs, both of which are well-known to disrupt the removal of damaged tissue and the collective wound healing process (Anderson and Hamm, 2012; Wang et al., 2013).

### 4.4 Optimal treatment for stimulating cartilage /tendon growth in the knee with osteoarthritis

Knee osteoarthritis (KOA) commonly involves loss of supporting cartilage, with knee replacement surgery often being the usual recourse. Three out of three studies demonstrated a statistically significant increase in cartilage thickness after HILT compared with baseline, indicating that high-intensity laser therapy can stimulate cartilage regeneration (Akaltun et al., 2020; Ekici and Ordahan, 2023; Şen et al., 2025). By contrast, a 2017 study failed to show increased cartilage, suggesting that a **daily** treatment regimen is important for sustained recovery, including lasting pain reduction and true cartilage growth (Alayat et al., 2017). Similar powers (12 W in the positive study vs. 10.5 W in the negative), wavelengths (both 1,064 nm), and pulse settings (10 Hz vs, 30 Hz) were used in the positive studies, but the more successful protocols all delivered treatments 5 *consecutive* days per week for at least 2 weeks, versus 2 days per week for 6 weeks in the negative study (Akaltun et al., 2020; Şen et al., 2025). Overall, these results indicate that to maximize the chance of stimulating cartilage growth with sustained benefits, HILT should be delivered on a daily schedule to ultimately favor a net regeneration effect over daily wear and tear tissue loss.

Notably, the sizes of partial tear rotator cuff tears (PTRCT) were significantly decreased with a large effect after treating patients with hemiplegic shoulder pain using HILT compared to controls in a study performed by Korkmaz et al., which utilized 8 W/cm^2^ administered 3 times per week for 3 weeks (ES_*PTRCT*_ = 0.9; Korkmaz et al., 2022). Another study demonstrated that HILT treatment (5 W/cm^2^) effectively reduced inflammation of supraspinatus tendons in patients with subacromial impingement syndrome (Zaki et al., 2022). These results are indicative that the high-powered laser can stimulate tendon growth.

### 4.5 Emerging consensus on knee laser PBMT

Based on the summary data, knee arthropathies and injuries appear poised to be the next indications most likely to be recognized in consensus guidelines and covered by insurance companies. This indication has the largest body of controlled trial data with generally consistent positive outcomes. Not only does knee arthropathy respond favorably to high-powered laser PBMT, as concluded from this meta-analysis, but the American Academy of Orthopedic Surgeons (AAOS) now recommends laser therapy in its latest recommendations for the non-arthroplasty treatment of osteoarthritis of the knee joint to “improve pain and function.”(American Academy of Orthopedic Surgeons, 2021) Investigators point out that patients should expect to experience improvements in pain and function, and significantly, there have been no reports of serious side effects from laser treatment for pain control (Prweb, 2022 AAOS Now Including Laser Therapy as a Treatment Guideline for Osteoarthritic Knee Pain, n.d.). A 2022 meta-analysis focused on laser PBMT for treating KOA reached the same conclusion: summary data indicates high-powered laser PBMT works for relieving pain and stiffness in KOA, especially when combined with exercise therapy and using higher powers (Ahmad et al., 2022).

### 4.6 From modest LLLT results to clinically meaningful weight and respiratory improvements: systemic advantages of HILT

High-intensity laser therapy appears markedly more effective for clinically meaningful fat loss in people with obesity than does LLLT, which has demonstrated mixed, often modest to negative results (Sun et al., 2025). Unlike most LLLT trials, which typically show small, localized circumference changes with negligible effects on overall weight or metabolic risk, the single Class 4 HILT study in obese patients produced clear, statistically significant reductions in body weight, BMI, and waist circumference, together with a large Cohen’s *d* improvement in forced vital capacity (FVC; Abdelaal, 2023).

Because reduced lung function independently predicts cardiovascular events, incident diabetes, and all-cause mortality in obesity, concomitant gains in FVC indicate that high-intensity 1,064 nm protocols may confer broader cardiorespiratory and cardiometabolic benefits than localized fat reduction alone. When viewed against the largely cosmetic or modest metabolic changes reported in LLLT obesity trials, these broad systemic effects support that adequately powered HILT regimens may shift photobiomodulation from peripheral “shaping” toward a genuinely disease-modifying strategy for obesity-related respiratory and cardiometabolic risk, with implications for protocol selection, patient counseling, and future trial designs that should routinely incorporate spirometric endpoints alongside anthropometric outcomes.

### 4.7 High-intensity laser therapy for brain pathologies: early successes

While only a single controlled trial has directly targeted the brain, as summarized in this meta-analysis (parameters: 3.4 W, 1,064 nm), an expanding body of exceptional case reports and series underscores the potential of HILT and related tPBM approaches across diverse brain pathologies (Han et al., 2025). Table 6 summarizes these studies. These include reversal of prosopagnosia after qEEG-guided transcranial laser therapy in a patient with temporal lobe epilepsy and mild cognitive impairment (parameters: 5–15 W, 810 nm). They also include chronic TBI in Veterans receiving pulsed tPBMT with LEDs that improved neuropsychological performance and regional cerebral blood flow (parameters: 13.2 W of 810 nm or 9 W of 810 nm + 980 nm). Additional evidence comes from TBI with comorbid depression and possibly primary major depression treated with multi-Watt NILT, which showed high response and remission rates with durable benefit (parameters: 8–15 W, 810 nm + 980 nm). Generalized anxiety disorder has also improved with self-administered NIR t-PBM, with significant reductions in anxiety and better sleep quality (parameters: 2.4 W, 830 nm). Collectively, these brain-directed studies, detailed in Table 6 (Morries et al., 2015; Henderson and Morries, 2017; Maiello et al., 2019; Hedaya and Lubar, 2022), together with the rising global burden of Alzheimer’s disease and related dementias and the absence of established effective treatments, strongly support further rigorous research into the broader therapeutic potential of HILT for brain pathologies.

**TABLE 6:** Clinical and neurophysiological outcomes of transcranial high-intensity laser therapy HILT and related photobiomodulation protocols for neuropsychiatric and cognitive disorders.

References	Study type	λ (nm)	Power	Settings	Treatment plan	Outcome
Prosopagnosia (Hedaya and Lubar, 2022)	Case study	810	25 W 3.5 W/cm^2^	5–60 J/cm^2^ 1,100–4,000 J/area QEEG-dependent focus	3×/wk 25× total FU: 1 mo	Cognitive improvement reduction in seizures QEEG improved
Generalized anxiety disorder (Maiello et al., 2019)	*n* = 12	980	2.4 W	30 mW/cm^2^ 20 m 36 J/cm^2^ 2.9 Kj per session	6×/wk 8 wks FU: EOT, 5, and 8 wks	improvement in sleep reduction in hamilton anxiety scale
Traumatic brain injury (Hipskind et al., 2019)	*n* = 12	850	3.3 W	180 LEDs, 6 mW/cm^2^ 75, 587, and 1,175 Hz; authors stressed importance of pulsing 20 m	3x/wk 6 wk FU: 3 wk	Increased blood flow measured by SPECT improvements in neuropsychological scores
Depression co-morbid with traumatic brain injury (Henderson and Morries, 2017)	*n* = 39	810 980	8–15 W	50–80 J/cm^2^ 9–12 m	8–34 treatments FU: 2, 6, 12, and 55 mos (5 cases for 55 mos)	The first report showing efficacy for depression. benefits ≤ 4 treatments. resolution for some ≤ 4 weeks.
Traumatic brain injury (Morries et al., 2015)	*n* = 10	810 (13W) or 810 + 980 (9W)	13 W 9 W	55–88 J/cm^2^ 2,400–3,000 J	10×/2 mos FU: EOT and 2 wks	Sleep improved Suicidal ideation reduced
Vascular dementia and schizophrenia (Hedaya and Carnahan, 2021)	2 case studies	Quantitative electroencephalography-directed transcranial HILT (similar to Hedaya; Hedaya and Lubar, 2022) Visual distortions and reading improved in 24y old paranoid schizophrenic after 4 treatments Dramatic improvements in memory in 65 y old vascular dementia patient after 20 treatments				
Multiple studies (Tian et al., 2016; Wang et al., 2017, 2021; search: transcranial photobiomodulation enhances visual working—NLM, 2024)	Demonstrable increases working memory, increases in cerebral oxygenation, increased cytochrome C oxidase, and increased alpha and beta brain waves as quantified by electroencephalography (EEG).					

FU, follow-up; EOT, end of treatment plan follow-up.

### 4.8 Toward precision, photon-based photobiomodulation dosing

The future of photobiomodulation depends on being able to measure and tailor the light “dose” with the same precision we expect from drug dosing. Instead of only describing treatments in terms of power (milliwatts) or energy delivered over time (joules), dosing can be framed in terms of the actual number of photons that reach the tissue. Using a photon-based unit such as the “Einstein” (one mole of photons, *6.0210^23^* photons) provides a fundamental measure that is independent of the specific device design and easily comparable across studies. Because this approach applies equally to both laser and LED sources, it offers a common language for dose reporting and optimization. As photon-based quantification methods become more practical in clinical and research settings, they will support truly personalized PBMT, allowing clinicians to adjust parameters so that each patient receives a biologically meaningful photon dose tailored to their tissue properties, condition, and treatment goals.

### 4.9 Study limitations

This systematic review and meta-analysis has several important limitations. First, one co-author (C.E.V.) is CEO of Aspen Laser and a named inventor on patents related to high-intensity laser therapy devices, representing a significant financial conflict of interest. Although transparently disclosed, this may introduce bias in framing the clinical relevance of high-intensity laser therapy, emphasizing favorable findings, or interpreting equivocal results.

To minimize this influence, the protocol (including PICO, eligibility criteria, and analytic plan) was prospectively registered in PROSPERO before screening began, and the search strategy and initial screening were led by the non-industry author (W.T.P.) using PRISMA 2020 and Cochrane guidance across multiple databases without manufacturer restrictions. Full-text eligibility decisions, data extraction, and risk-of-bias assessments followed pre-specified criteria and standardized forms, with the industry-affiliated author excluded from trial inclusion decisions and risk-of-bias ratings. Quantitative synthesis used conventional random-effects methods based solely on published trial data, and we highlighted heterogeneity, small sample sizes, and other methodological limitations. Nevertheless, residual bias related to financial interests cannot be fully excluded, particularly in interpretive emphasis and discussion of implications for practice and reimbursement.

Our study had additional limitations related to the evidence base. Other than one knee arthropathy trial using 30 W, the highest-powered laser in the included RCTs was 15 W, whereas class IV devices up to 80 W are already in clinical use but remain untested in controlled trials. We found no RCTs evaluating average powers above 30 W. Most studies also used the 1,064 nm wavelength; higher-powered trials at other wavelengths within the 650–830 nm range, particularly 810–830 nm, which is minimally absorbed by water and thus better suited for deeper, lower-heat PBMT, are lacking.

Additional limitations include heterogeneous study designs, variable follow-up durations, frequent failure to report spot size (precluding accurate power-density estimates), and small sample sizes, all of which may limit generalizability and introduce publication bias. Finally, treatment targeting may have influenced outcomes. For some conditions, lasers may not have been directed toward the true source of pathology or along relevant neural pathways. For example, hip stretching and strengthening can relieve distal foot pain, suggesting that proximal interventions can modulate distal symptoms; analogously, for referred extremity pain, targeting the spinal cord or central nervous system might yield greater benefit than treating only peripheral sites.

## 5 Conclusion

This systematic review and meta-analysis of 105 controlled clinical trials provides robust, high-level evidence that power is critical for successful photobiomodulation in deep-tissue pathologies. Class IV high-intensity laser therapy (HILT), using average powers of 9.3 W (0.5–80 W) and power densities up to 52.5 W/cm^2^, consistently delivers therapeutic photon doses to depths of 3–15 cm, with large clinical effect sizes across domains where low-level laser therapy (LLLT) has historically underperformed.

LLLT remains valuable for superficial conditions (< 2 cm) and is supported by extensive foundational research, but many earlier LLLT trials for deeper musculoskeletal and neurological disorders used low-power, outdated devices unable to achieve adequate penetration. These studies often emphasized safety while yielding modest, inconsistent results and paid limited attention to protocol optimization. In contrast, HILT showed highly consistent efficacy: statistically and clinically significant pain reduction in 100% of randomized trials for knee (*k* = 27), spine/neuromuscular (*k* = 20), and shoulder/arm (*k* = 20) conditions, with over 90% reporting large effects (Cohen’s *d* > 0.8). Benefits were durable, with sustained large VAS pain reductions at ≥ 3 months and extended to range of motion (95% of studies), functional performance (84%), quality of life (85%), and structural outcomes such as increased cartilage thickness, reduced rotator cuff tear size, and improved nerve conduction. HILT outperformed LLLT in all 13 head-to-head studies, with no serious adverse events.

These findings support a pragmatic, evidence-based approach: Class III (LLLT) and Class IV (HILT) lasers have complementary roles. LLLT suits superficial indications where minimal thermal effects are desired, whereas HILT should be preferred when deeper penetration and robust energy delivery are needed. Protocols should be individualized to anatomical depth, pathology, and patient response. The future of photobiomodulation lies in precise, personalized dosimetry; photon-based metrics such as the Einstein unit (one mole of photons, 6.0210^23^), applicable to both lasers and LED, may further refine dosing and treatment optimization.

Clinicians and healthcare providers are encouraged to incorporate these findings into practice. HILT offers a safe, effective, non-invasive option that can reduce reliance on pharmacologic therapies, support tissue healing, improve function, and lower long-term healthcare costs. By moving from class-based debates toward physics-informed, outcome-driven decision-making, the medical community can more fully realize the therapeutic potential of photobiomodulation in challenging deep-tissue conditions. This report provides a strong foundation for expanded adoption, guideline development, and appropriate payer coverage of HILT as a valuable addition to modern musculoskeletal and regenerative medicine.

## Data Availability

The datasets presented in this study can be found in online repositories. The names of the repository/repositories and accession number(s) can be found in the article/Supplementary material.
