# Correction: The importance of power in photobiomodulation: a systematic review and meta-analysis of high-intensity laser therapy

**DOI:** 10.3389/fnint.2026.2005993

**Published:** 2026-10-19

**Authors:** William Todd Penberthy, Charles E. Vorwaller

**Affiliations:** 1 Personalized Health Research Services, LLC, Winter Park, FL, United States; 2 Aspen Lasers, LLC, Lindon, UT, United States

**Keywords:** photobiomodulation therapy, high-intensity laser therapy, class IV laser therapy, transcranial photobiomodulation, laser power, musculoskeletal pain, systematic review and meta-analysis, low-level laser therapy

In the published article, an **Acknowledgment** recognizing two non-author contributors was erroneously omitted. The following **Acknowledgments** section has been added to the article.

The original version of this article has been updated.

