# Supplementary material for: The importance of power in photobiomodulation: a systematic review and meta-analysis of high-intensity laser therapy
Source: Front Integr Neurosci. 2026 Sep 14;20:1914223. doi: 10.3389/fnint.2026.1914223 (PMC13625623; doi:10.3389/fnint.2026.1914223)
Supplement: Supplementary file 1 [file Data_Sheet_1.pdf]

Study	1	2	3	4	5
Abdel-aal; hand osteoarthritis in SLE; 2020	+	+	+	X	+
Abdelbasset; lower back pain; 2020	+	X	X	X	X
Abdelhakiem; hemiplegic shoulder; 2024	+	+	X	X	X
Abdelmageed; chronic discogenic sciatica; 2022	+	+	X	X	-
Abdullatif; Bell's palsy; 2022	X	-	X	X	-
Aceituno-Gómez; subacromial impingement; 2019	+	+	+	X	+
Ahi; De Quervain's tenosynovitis; 2023	+	+	+	X	+
Ahi; myofascial pain syndrome; 2022	+	X	X	X	+
Ahmad; knee osteoarthritis; 2023	+	+	+	X	+
Akaltun; knee osteoarthritis; 2020	+	+	X	X	+
Al-sharani; periodontitis; 2026	+	?	X	X	+
Alayat; osteoporosis; 2018	+	-	X	X	+
Anupma; knee osteoarthritis; 2025	+	?	+	X	+
Atan; adhesive capsulitis; 2021	+	+	+	X	+
Bettancourt; knee osteoarthritis; 2020	+	+	+	X	-
Bidoki; plantar fasciitis; 2024	+	+	X	X	X
Cantero-Tellez; thumb osteoarthritis; 2020	+	X	+	+	+
Ceyhan; KOA; 2025	+	+	+	X	X
Chatterjee; diabetic peripheral neuropathy; 2019	+	+	+	+	+
Chen; lumbar disc protrusion; 2018	+	X	X	X	X
Chongkriengkrai; de Quervain's tenosynovitis; 2023	+	+	+	X	+
Ekici knee osteoarthritis; 2023	+	+	+	X	+
Ekici; myogenic temporomandibular joint disorder; 2021	+	+	+	X	+
El-Shamy; hemophilic arthropathy; 2018	+	+	+	X	X
El-Shamy; juvenile rheumatoid arthritis; 2018	+	+	+	X	+
Elkallah; chronic sinusitis; 2020	+	-	X	?	?
Elnagger; hemophilic A ankle arthropathy; 2020	+	-	+	+	X
Elsodany; rotator cuff tendinopathy; 2018	+	-	+	X	+
Ezzati; carpal tunnel syndrome; 2020	+	X	+	X	+
Ezzati; knee osteroarthritis; 2025	+	-	+	X	-
Gawaan; myofascial pain syndrome post-neck; 2023	+	-	X	X	-
Gungor; Calcaneal spur; 2025	+	+	+	X	X
Han; mild cognitive impairment; 2025	+	+	+	X	X
Ince; neck pain; 2024	+	-	X	X	+
Jitpimolmard; plantar fasciitis; 2026	+	?	+	X	+
Karaca; lateral epicondylitis; 2022	+	-	X	X	X
Kaydock; lateral epicondylitis; 2020	+	+	X	X	X
Kelini; fibromyalgia; 2025	+	+	+	X	X
Kenareh; chronic neck pain; 2021	+	+	X	X	+
Ketz; plantar fasciitis; 2024	+	?	X	X	X
Khoramdel; chronic regional pain syndrome; 2025	+	+	X	X	+
Koevska; KOA; 2025	+	+	X	X	+
Korkmaz; hemiplegic shoulder pain; 2021	+	+	X	X	+
Kumar; coccydynia; 2025	X	X	X	X	X
Laotammateep; KOA; 2025	+	+	+	X	+
Lu; chronic refractory wounds; 2021	+	+	X	X	+
Mostafa; knee osteoarthritis; 2022	+	+	X	X	+
Nadershah; temporomandibular joint disorder; 2020	+	-	+	X	+
Naruseviciute; plantar fasciiitis; 2020	+	-	+	X	-
Nazari; knee osteoarthritis; 2019	+	X	X	+	X
Nouri; patellofemoral pain syndrome; 2019	+	-	+	X	X
Ordahan; adhesive capsulitis; 2023	+	+	+	X	+
Ordahan; plantar fasciiitis; 2018	+	+	+	X	+
Ozgozen; KOA; 2025	+	+	+	X	+
Saleh; subacromial impingement syndrome; 2025	+	+	+	X	+
Saman; knee osteoarthritis; 2022	+	-	X	X	-
Sen; lateral epicondylitis; 2024	+	+	X	X	X
Shabaan; COVID19 ageusia; 2023	+	+	+	X	+
Sherif; cervical radiculopathy; 2023	+	-	X	-	-
Siriratna; knee osteoarthritis; 2022	+	+	+	X	+
Song; KOA; 2025	+	+	+	X	X
Taheri; knee osteoarthritis; 2024	+	-	X	X	X
Tangsriwong; KOA; 2025	+	+	+	X	+
Thabet; caesarian healing in diabetic women; 2018	+	-	X	X	X
Thabet; endometriosis; 2018	+	-	+	X	+
Uzun; supraspinatus tears; 2025	+	+	+	X	X
Venosa; cervical spondylosis; 2019	+	-	X	X	X
Vetrici; COVID19; 2021	+	+	X	X	+
Wibisono; knee osteoarthritis; 2024	+	-	X	?	?
Wu; Bell's palsy; 2023	+	-	X	X	X
Yesil; calcaneal spur; 2019	+	X	+	X	+
Yilmaz; cervical disc herniation; 2020	+	-	X	X	-
Yilmaz; subacromial Impingement Syndrome; 2021	+	+	+	X	+
Yuksel; lateral elbow tendinopathy; 2025	+	+	+	X	X
Zaki; subacromial impingement syndrome; 2022	+	+	X	X	-
Indolia; post-stroke limb mobility; 2024	+	?	X	X	-
Jangra; acute doronary syndrome; 2024	+	+	X	X	+
Elyazed; hemiparetic osteoparetic stroke; 2023	+	+	+	X	+
Xie; neck pain; 2025	+	+	+	X	+
Natalia; knee osteoarthritis; 2024	+	+	+	X	+
Royheym; knee Osteoarthritis; 2023	+	+	+	X	+
Ciplak; knee osteoarthritis; 2018	+	+	?	?	?
Tkocz; calcineal spur with plantar fasciitis; 2021	+	X	+	+	X
Ashour; carpal tunnel syndrome; 2022	+	+	X	X	+
Astri; knee osteoarthritis; 2023	+	+	+	?	?
Yadav; plantar fasciitis; 2026	+	+	+	X	+
Abdelaal; abdomen; 2026	+	+	+	X	+
Shrestha; chronic neck pain; 2024	+	+	X	X	+
Qataya; temporamandibular disorder; 2025	+	+	X	X	+
Hojjati; carpal tunnel syndrome; 2020	+	+	X	X	+
Rahbar; myofascial trigger points; 2025	+	+	X	+	+
Rizki; knee osteoarthritis; 2025	+	+	?	?	?
Elerian; diabetic frozen shoulder; 2024	+	+	X	X	+
Dajpratham; hemiplegic shoulder pain; 2025	+	+	X	+	+
Ahmed; myofascial trigger points; 2020	+	?	?	?	?

1. Random sequence generation (selection bias)
2. Allocation concealment (selection bias)
3. Blinding of participants (performance bias)
4. Blinding of personnel (performance bias)
5. Blinding of outcomes assessment (detection bias)
