# Supplementary material for: The importance of power in photobiomodulation: a systematic review and meta-analysis of high-intensity laser therapy
Source: Front Integr Neurosci. 2026 Sep 14;20:1914223. doi: 10.3389/fnint.2026.1914223 (PMC13625623; doi:10.3389/fnint.2026.1914223)
Supplement: Supplementary file 2 [file Table_1.docx]

**Supplementary table S1.** Effect size Cohen’s *d* calculations are shown.
